# Polarized luminescence in halide perovskite nanocrystals: from fundamental microscopic mechanisms to advanced optoelectronic applications

**DOI:** 10.3389/fchem.2026.1945484

**Published:** 2026-09-01

**Authors:** Jinfei Dai, Yanni He, Yingrui Luo, Wenxi Gao, Canpu Yang, Hassan Muhammud, Fatima Ghazala, Jie Xu, Fang Yuan

**Affiliations:** 1 Key Laboratory for Physical Electronics and Devices of the Ministry of Education & Shaanxi Key Lab of Information Photonic Technique, School of Electronic Science and Engineering, Xi’an Jiaotong University, Xi’an, China; 2 School of Materials Science and Engineering, Xi’an Jiaotong University, Xi’an, China; 3 School of Science, Xi’an University of Architecture and Technology, Xi’an, China

**Keywords:** chiral ligands, halide perovskite nanocrystals, optoelectronic devices, polarized assembly, polarized luminescence

## Abstract

Polarized luminescent perovskite nanocrystals (PNCs) have emerged as promising optoelectronic materials due to their high photoluminescence quantum yield, narrow emission linewidth, and excellent structural tunability. Benefiting from unique structural anisotropy, chiral ligand modulation and stimulus responsiveness, PNCs enable efficient linear and circular polarized luminescence. This review systematically elaborates the intrinsic mechanisms of PNC-based polarized emission, summarizes advanced macroscopic assembly and structural engineering strategies for stabilizing film state polarized luminescence, and outlines state-of-the-art applications in optoelectronic devices, encryption and spintronics. The current bottlenecks and future development perspectives are also proposed, aiming to promote the advancement of polarized perovskite optoelectronics.

## 1 Introduction

Polarization represents one of the most fundamental and distinctive physical properties of light, describing the directional oscillation of the electric-field vector during optical propagation. Natural unpolarized light features randomly distributed electric-field vibrations perpendicular to the propagation direction, whereas polarized light originates from the superposition of two orthogonal monochromatic waves with identical frequencies. Variations in the phase difference between these orthogonal components give rise to two dominant polarization states, linear polarization and circular polarization. Owing to their ordered and anisotropic optical characteristics, polarized light endows optoelectronic systems with unique technical advantages that are inaccessible for conventional unpolarized light sources. In advanced display technologies, linearly polarized emission enables precise orientation matching with liquid-crystal molecules, effectively improving the backlight utilization efficiency of liquid-crystal displays (LCDs) and reducing overall power consumption (Cunningham et al., 2016; Ge et al., 2019; Zhang et al., 2023). For stereoscopic 3D displays, orthogonal polarization splitting suppresses optical crosstalk and significantly enhances imaging fidelity (Wu et al., 2023; Zhang et al., 2024b). Beyond display applications, polarization-resolved photodetectors are capable of discriminating subtle polarization-state variations of incident light, greatly improving signal recognition and anti-interference capability in complex optical environments (Long et al., 2020). Such unique superiority renders polarized optoelectronic technologies highly promising for extensive applications in remote sensing, biomedical imaging, and high-security quantum information encryption (Hou et al., 2022; Lu et al., 2025; Peng et al., 2021).

As an emerging class of solution-processable optoelectronic materials, perovskite nanocrystals (PNCs) have attracted intensive research attention due to their superior and tunable photophysical properties (Dai et al., 2024; Guo et al., 2026; Li et al., 2023; Yang Z. et al., 2024). In comparison with traditional colloidal semiconductor nanocrystals (e.g., CdSe-based quantum dots), PNCs exhibit prominent competitive advantages, including near-unity photoluminescence quantum yields (PLQY), ultranarrow emission linewidths (full-width at half-maximum, FWHM below 20 nm), and ultrabroad spectral tunability covering the entire visible region (400–800 nm). More importantly, PNCs possess inherent structural and photophysical peculiarities that make them ideal platforms for polarized luminescence modulation. First, the rich surface coordination sites of PNCs support efficient binding and interfacial coupling with chiral organic ligands, enabling effective chirality transfer from molecular stereo-structures to inorganic perovskite frameworks. Second, their versatile low-dimensional crystal architectures enable facile structural anisotropy engineering, allowing direct generation of intrinsic linear polarized emission via morphology tailoring or lattice distortion regulation (Prasanna et al., 2017; Täuber et al., 2016). Third, PNCs exhibit excellent stimulus responsiveness toward electric fields, magnetic fields, temperature, and mechanical strain, facilitating macroscopic ordered alignment and polarization optimization of nanocrystal assemblies (Hou et al., 2022; Täuber et al., 2016; Wang X. et al., 2021). Benefiting from the above comprehensive merits, PNC-based polarized luminescent materials have emerged as promising candidates for next-generation high-efficiency polarized optoelectronic devices (Fu et al., 2017). Accordingly, this article comprehensively reviews the current research status of optoelectronic devices based on polarized perovskite nanocrystals.

This review systematically summarizes the state-of-the-art progress of polarized luminescence research based on perovskite nanocrystals. We first elaborate the fundamental physical mechanisms of linear and circular polarized emission in PNCs. Subsequently, we comprehensively classify macroscopic assembly and structural engineering strategies for preserving linear and circular polarized luminescence of perovskite nanocrystals in aggregated thin-film states. On this basis, we overview the cutting-edge applications of PNC-based polarized optoelectronic systems in light-emitting devices, photodetectors, optoelectronic encryption systems, and spintronic devices. Finally, we highlight the core technical bottlenecks restricting the practical translation of polarized perovskite devices and provide insightful perspectives on future development directions, aiming to offer a systematic and comprehensive reference for the advancement of perovskite polarized optoelectronics.

## 2 Polarization luminescence mechanism of perovskite materials

Perovskite materials can generate highly polarized luminescence, whose physical origin lies in the optical anisotropy exhibited by the material at both nanoscale and macroscale levels. This anisotropy stems from the intrinsic crystal structure and electronic state properties of the material and can also be achieved and enhanced through external assembly and control strategies. This chapter will systematically elaborate on the core physical mechanisms of perovskite polarized luminescence.

### 2.1 Linearly polarized emission mechanisms

According to the dipole theory of electromagnetic oscillation, the essence of directional light emission is governed by the magnitude and orientation of the transition dipole moments (TDMs) during electron-hole pair recombination (Marcato et al., 2022). In perovskite materials, various intrinsic and extrinsic structural factors can induce the alignment of TDMs, thereby yielding linearly polarized emission. These anisotropies primarily originate from the following dimensions.

#### 2.1.1 TDM reoriented from intrinsic lattice distortion

**FIGURE 1:**
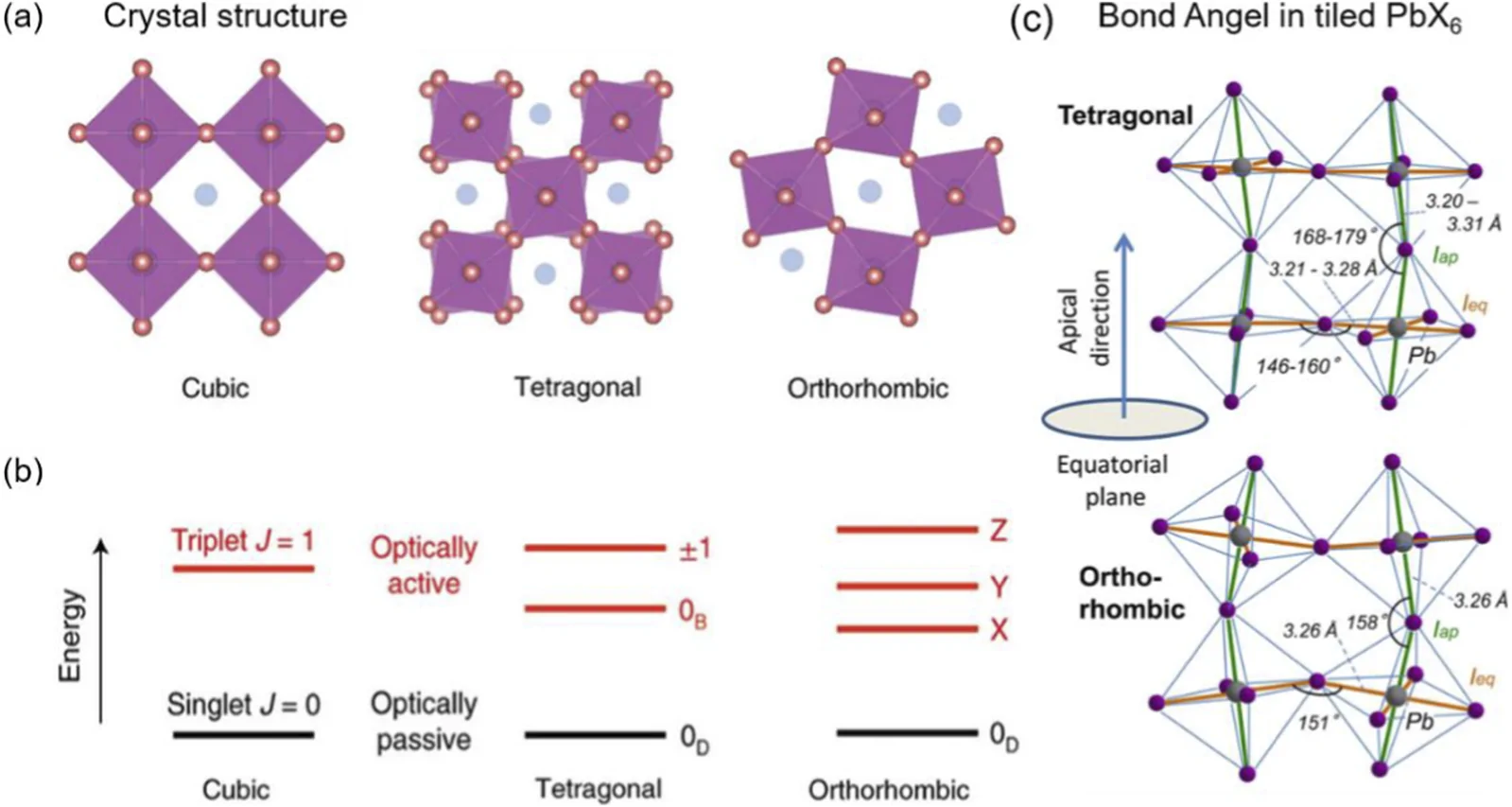
Intrinsic lattice origins of linearly polarized luminescence in perovskites. **(a)** Crystal lattice structures of perovskites with different crystalline phases; **(b)** Excitonic energy level alignment in various perovskite phases; **(c)** Octahedral linkage and distortion behaviors in tetragonal and monoclinic perovskite phases. Copyright 2018, Royal Society of Chemistry (Ramade et al., 2018).

The PbX_6_ octahedral framework constitutes the fundamental structural unit of halide perovskites and primarily governs their band-edge electronic properties. The long-range periodic assembly and local tilting distortion of PbX_6_ octahedra collectively modulate the electronic band structure by breaking the structural symmetry of the lattice. Figure 1a schematically presents the distinct topological configurations of PbX_6_ octahedra in three typical perovskite phases: cubic, tetragonal, and orthorhombic lattices. The structural transition from high-symmetry cubic phase to low-symmetry tetragonal and monoclinic phases induces gradual symmetry breaking, which lifts the degeneracy of exciton states and yields discrete exciton fine-structure sublevels as shown in Figure 1b. These fine-structure sublevels exhibit anisotropic transition orientations and disparate radiative recombination probabilities, which redistribute the orientation of TDMs within the crystal lattice. Consequently, such symmetry-originated TDMs anisotropy endows low-symmetry perovskites with characteristic linearly polarized photoluminescence. Using high-resolution photoluminescence spectroscopy, Ramade et al. discovered that the exciton fine structure in CsPbBr_3_ nanocrystals exhibits a series of linearly polarized sharp peaks, which assemble in pairs to form distinct doublets or triplets (Ramade et al., 2018). What’s more, the intrinsic structural distortion of PbI_6_ octahedra lifts the degeneracy of excited and creates energy splitting, Figure 1c display the Pb-I angel difference in equatorial and apical direction of tetragonal, and orthorhombic phase of MAPbI_3_. Owing to the difference of bond angles in different direction, and the bandgap depends on the angle, therefore the transition dipole of the lowest optical transition is fixed along the crystal apical Pb-I-Pb axis, the material exhibits strongly polarized photoluminescence.

#### 2.1.2 TDM modulate by shape anisotropy of nanocrystal

**FIGURE 2:**
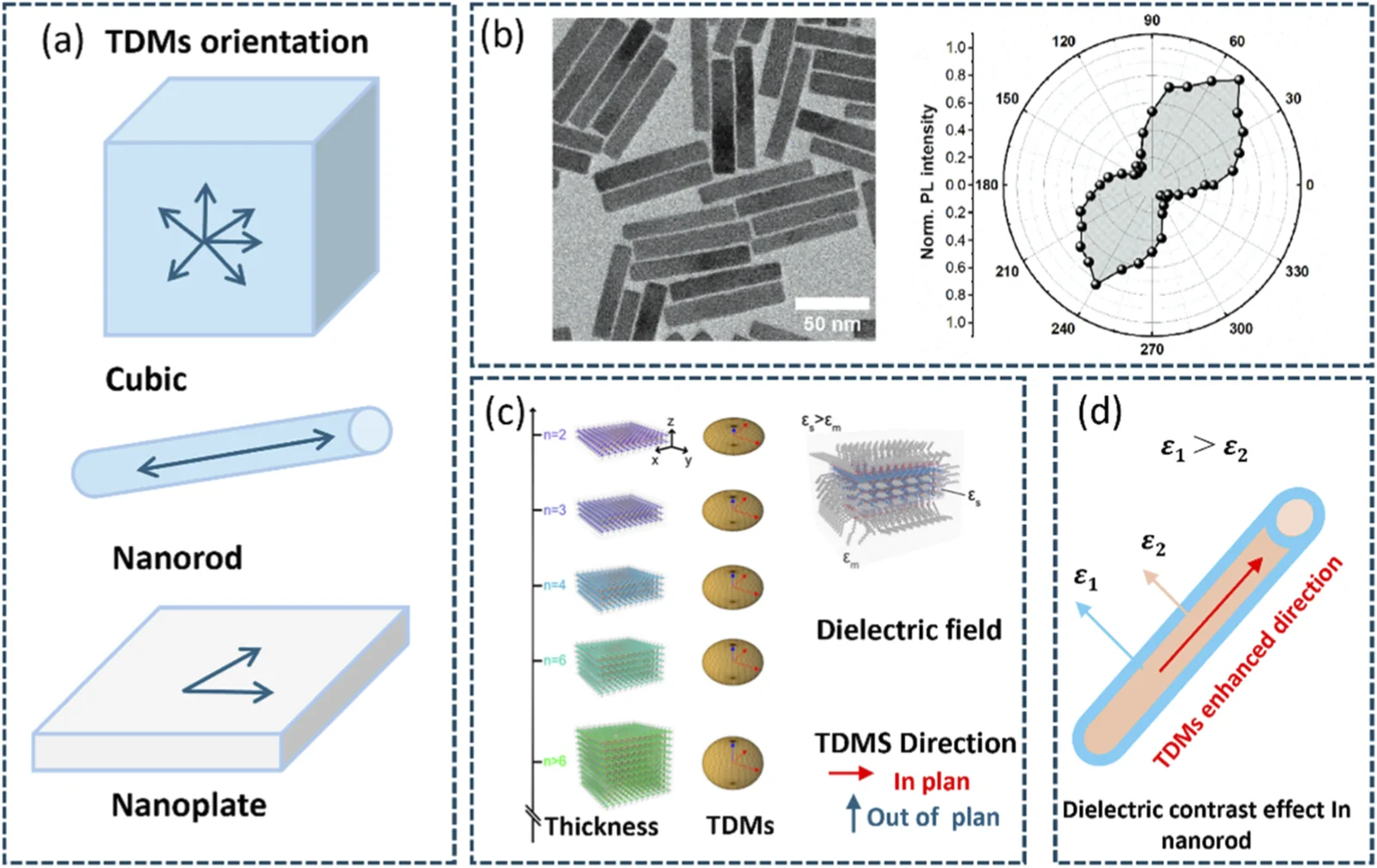
Effects of morphological and dielectric anisotropy on transition dipole orientation and polarized luminescence. **(a)** Transition dipole orientations in perovskite nanocrystals, nanorods, and nanoplatelets; **(b)** Morphology and polarized luminescence performance of CsPbBr_3_ nanorods with an aspect ratio of 6.5, Copyright 2026 Tsinghua University Press (Toperczer et al., 2026); **(c)** Transition dipole variations induced by octahedral layer thickness and environmental dielectric effects in nanoplatelets, Reproduced with permission (Sekh et al., 2026); **(d)** Polarized emission behavior of nanowires modulated by dielectric contrast effects.

Beyond intrinsic lattice distortion, morphological anisotropy of low-dimensional perovskite nanocrystals further modulates exciton wavefunction distribution and TDM orientation via dimensional quantum confinement. The confinement strength varies drastically between long and short dimensions of anisotropic nanostructures, redistributing electron-hole orbital overlap and lifting the degeneracy of triplet exciton fine states, thereby locking the ground-state exciton subband and corresponding TDM along the direction of weakest quantum confinement, as illustrated in Figure 2a. For one-dimensional perovskite nanowires and nanorods, geometric anisotropy induces distinct dimensional confinement effects. Specifically, perovskite nanowires undergo strong quantum confinement along both radial lateral axes, restricting the spatial extension of exciton wavefunctions in the cross-sectional plane; by contrast, the ultra-long axial dimension exhibits negligible confinement, allowing exciton wavefunctions to elongate freely along the wire growth direction. Such asymmetric confinement strongly orients the ground-state TDMs parallel to the nanowire long axis, yielding linearly polarized photoluminescence polarized along the axial direction. Similarly, perovskite nanorods possess size-dependent radial confinement and unconstrained longitudinal extension, with TDMs preferentially aligned along the rod long axis to produce long-axis polarized emission. Toperczer et al. systematically investigated the polarized emission characteristics of single CsPbBr_3_ nanorods with an average aspect ratio of approximately 6.5 (Toperczer et al., 2026). These nanostructures featured a typical length of 81 nm and a thickness of 12.5 nm Polarization-resolved measurements revealed that the emission intensity followed a perfect cosine-squared dependence as a function of the analyzer angle as shown in Figure 2b, demonstrating that the TDM is strictly oriented along the long axis of the nanorods.

In contrast, two-dimensional perovskite nanocrystals are only several monolayers thick, a dimension much smaller than the exciton Bohr radius, whereas their lateral sizes span tens to hundreds of nanometers. Such pronounced geometric anisotropy generates robust in-plane quantum confinement, which locks most TDMS within the nanoplatelet plane and produces photoluminescence polarized parallel to the platelet surface. Importantly, the orientation and strength of these confined transition dipoles can be readily tuned by adjusting the layer count of 2D perovskite nanocrystals, Figure 2c left. For perovskite nanoplatelets (e.g., CsPbBr_3_), Huo et al. (Sekh et al., 2026) observed exciton fine-structure splitting at cryogenic temperatures. Polarization-resolved spectroscopy further revealed that emission peaks at distinct energies exhibit nearly orthogonal polarization directions, solidifying the shape-induced anisotropy of TDMs. Sekh et al. synthesized highly monodisperse MAPbBr_3_ nanoplatelets (NPLs) via ligand engineering, where native oleylamine ligands were substituted with zwitterionic phospholipid or 2-ammonioethyl 2-octyl-1-dodecyl phosphate ligands. The as-synthesized NPLs possess lateral dimensions of 11.3 ± 2.3 nm and an ultrathin thickness of only 1.7 ± 0.5 nm (corresponding to ∼3 monolayers), accompanied by a high PLQY of 80%. More critically, polarization-resolved single-NPLs photoluminescence measurements performed at 4 K confirmed strongly linearly polarized emission, with degrees of polarization (DOP) reaching 88% and 81%, respectively. A sharp satellite peak was detected at an energy offset of 12.9 meV above the primary emission band, whose dipole orientation is nearly orthogonal to that of the main peak. This satellite peak is assigned to out-of-plane exciton fine-structure states.

#### 2.1.3 TDM anisotropy amplified by dielectric confinement

Perovskite nanocrystals feature large specific surface areas fully passivated by organic capping ligands. The stark dielectric mismatch between the high-permittivity inorganic perovskite core and low-dielectric ligand shell creates highly anisotropic local electric fields, an effect that becomes especially prominent in geometrically anisotropic nanocrystals. Complementing quantum confinement and lattice distortion, this core-ligand dielectric contrast acts as an extrinsic factor that amplifies the intrinsic orientational preference of TDMs.

Marcato et al. thoroughly probed how nanoplatelet layer thickness and dielectric anisotropy jointly regulate TDM alignment in 2D perovskite NPLs. Theoretical simulations confirm that the substantial permittivity offset between NPLs lattices (*ε* ≈ 4.7) and the surrounding low-dielectric ligand matrix (*ε* ≈ 2.1) produces a far larger local field factor along the in-plane direction than along the out-of-plane axis. The resultant dielectric confinement selectively boosts the radiative recombination rate of in-plane TDMs while quenching the radiative decay of out-of-plane dipoles, which further magnifies the shape-driven TDM anisotropy, Figure 2c. The dielectric contrast effect plays an even more dominant role in governing polarized emission from one-dimensional nanowires. As demonstrated by Johnston and Joyce (2022), dielectric mismatch stands as the primary source of polarization anisotropy for semiconductor nanowires with diameters ranging from 20 to 100 nm. This behavior originates from the extreme permittivity difference between the high-refractive-index nanowire core and its organic ligand coating. Electric field components parallel to the wire long axis propagate unimpeded through the crystalline core, whereas transverse field components are strongly screened by surface polarization charges, Figure 2d. This anisotropic dielectric confinement raises the effective oscillator strength of axially oriented TDMs, leading to linearly polarized photoluminescence aligned parallel to the nanowire growth direction.

### 2.2 Circularly polarized luminescence mechanisms of perovskite nanocrystal

Generally, the generation of circularly polarized luminescence (CPL) inherently requires the material system to possess chirality, which is defined as a geometric property wherein a structure is non-superimposable on its mirror image. According to the thermodynamics of perovskite crystallization, their lattices naturally crystallize into centrosymmetric or achiral space groups. Herein, the fundamental strategy for imparting chiroptical activity relies on breaking both inversion and mirror symmetries. This spatial symmetry breaking induces an asymmetry in the transition rates of left-handed and right-handed circularly polarized light, which ultimately results in circular emission.

In the field of PNCs, chiroptical activity primarily originates from three microscopic mechanisms (1) Chiral ligand-induced distortion of the surface lattice, (2) Electronic state coupling via orbital hybridization between the chiral ligands and the wavefunctions of inorganic perovskite core, (3) Intrinsic structural chirality incorporated directly into the nanocrystal core during nucleation and growth process. Mechanisms (1) and (2) represent extrinsic surface-driven chiral transfer approaches applicable to pre-synthesized achiral NCs, whereas mechanism (3) involves the direct design of intrinsically chiral perovskite NC architectures. This section systematically elucidates these strategies and establishes their physical foundations across multi-dimensional nanocrystal systems.

#### 2.2.1 Surface ligand-induced chiral transfer mechanism

Surface modification using chiral capping agents is the most straightforward and versatile strategy to endow pre-synthesized achiral perovskite NCs with robust CPL activity. Chiral organic molecules, such as R/S α-octylamine or R/S methylbenzylamine (R/S-MBA), coordinate to the NC surface via anchoring groups, transferring molecular chirality to the inorganic core through two main synergistic pathways. Those are surface lattice distortion mechanism and electronic state coupling mechanism.

##### 2.2.1.1 Extrinsic ligand-induced surface lattice distortion mechanism

**FIGURE 3:**
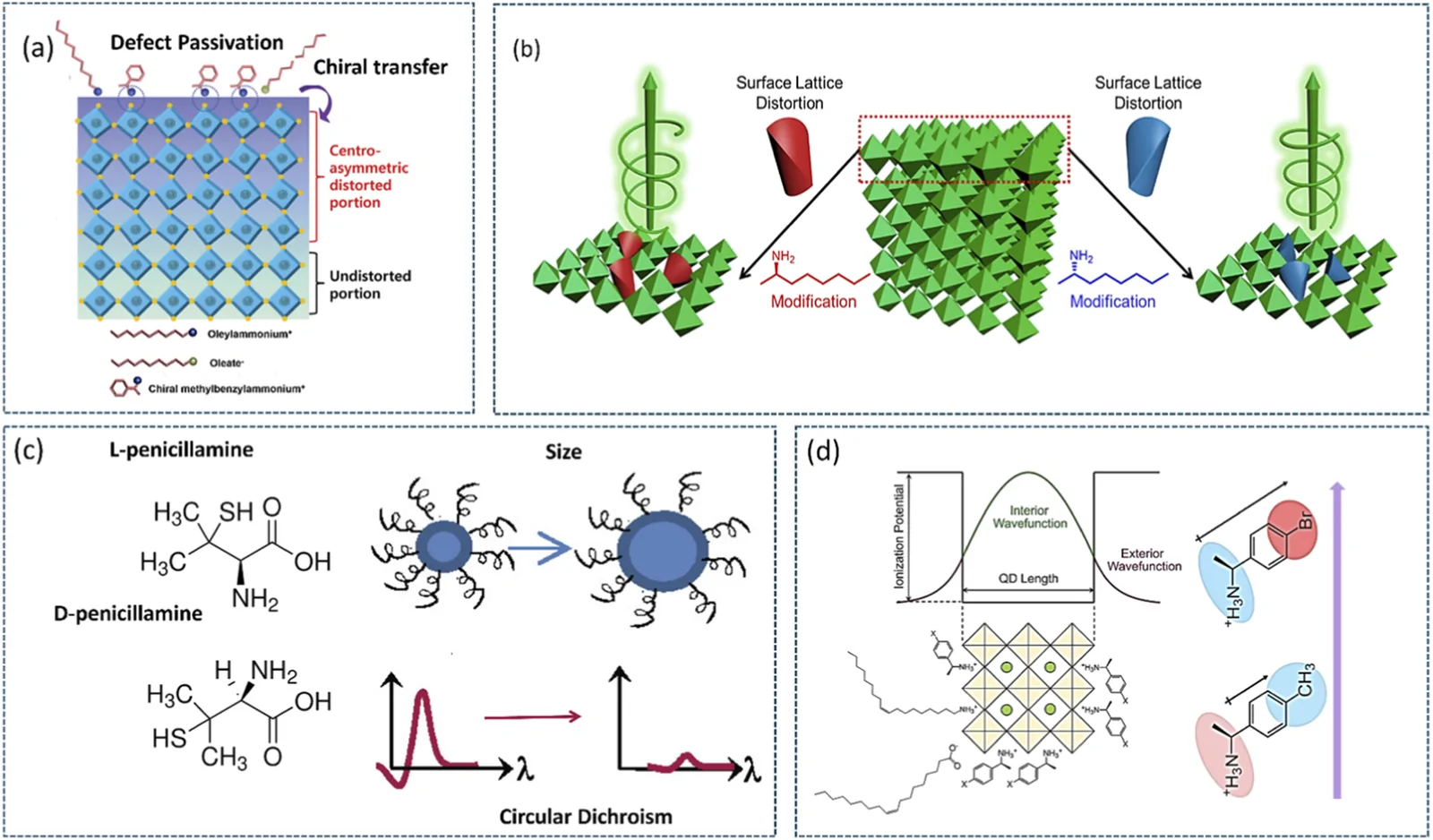
Chiral ligand-induced surface lattice distortion and electronic state coupling enabling chirality transfer in perovskite nanocrystals. **(a)** and **(b)** Chiral ligand binding to surface defects induces surface lattice distortion and subsequent chirality transfer, Copyright 2022, Wiley and 2019, American Chemical Society (Chen et al., 2019; Kim et al., 2022); **(c)** Size-dependent effects of nanocrystals on chiral ligand-nanocrystal electronic coupling and resultant circularly polarized emission; Copyright 2011, American Chemical Society (Ben Moshe et al., 2011). **(d)** Influence of chiral ligand electron distribution on interfacial electronic coupling and CPL performance, Copyright 2024, (Dunlap-Shohl et al., 2024).

Asymmetric bonding scenarios between the chiral ligands and surface atoms can propagate into the sub-surface region, forcing a chiral tilt in the inorganic framework. Kim et al. combined density functional theory calculations with spectroscopic validation to map the chiral transfer pathway in R/S MBA-capped CsPbBr_3_ perovskite NCs (Kim et al., 2022). They demonstrated that the stereospecific constraints of the ligand induce a non-centrosymmetric distortion of the surface [PbBr_6_] octahedra mediated by asymmetrical N-H-Br hydrogen bonding networks. Remarkably, this structural distortion penetrates up to five atomic layers deep into the NC core, effectively “imprinting” molecular chirality into the inorganic lattice and yielding prominent circular dichroism (CD) Figure 3a. Similarly, Chen et al. employed chiral α-octylamine to target surface Br^−^ vacancy sites on CsPbBr_3_ NCs (Chen et al., 2019). This localized asymmetric coordination created unique “chiral footprints” that broke the surface inversion symmetry of the otherwise achiral core, Figure 3b, enabling the first demonstration of two-photon absorption-based upconverted CPL (TPU-CPL).

To further illustrated this origin, He et al. conducted ligand-cleavage experiments combined with photophysical characterization on CsPb(I/Br)_3_ nanocrystals passivated with a trace amount of chiral 1,2-diaminocyclohexane, which is commonly abbreviated as DACH (He et al., 2017). Upon treating the nanocrystals with acetyl chloride to completely eliminate the surface chiral ligands, the remaining inorganic cores continued to display a CD signal at 247 nm that was nearly identical to that of the pristine high dense ligand-capped nanocrystals. The indicates that the surface lattice distortions induced by the chiral ligands are partially preserved even after ligand removal, thereby serving as the primary contributor to the lingering chiroptical signal in that specific ultraviolet region.

##### 2.2.1.2 Electronic state coupling mechanism

This pathway involves the direct wavefunction hybridization between the frontier molecular orbitals of the chiral ligands and the exciton states of the inorganic nanocrystals. Ben Moshe et al. established that chiroptical activity primarily arises from the partial delocalization and orbital hybridization of photogenerated holes into the molecular orbitals of the chiral ligands of penicillamine, which represents a direct electronic coupling between the ligand orbitals and the exciton wavefunction (Ben Moshe et al., 2011). This mechanism successfully accounts for both size-dependent and material-dependent chiroptical behaviors Figure 3c. Furthermore, it demonstrates that band-edge exciton transitions can inherit robust chiroptical activity specifically through such electronic interactions with the surface ligand matrix.

In a parallel advancement focusing on lower dimensions nanocrystal, Georgieva et al. reported a direct synthetic strategy to fabricate chiral methylammonium lead bromide (CH_3_NH_3_PbBr_3_) perovskite nanoplatelets, employing chiral R- or S-phenethylammonium (PEA) ligands combined with octylamine as co-ligands. The resulting nanoplatelets exhibited mirror-image CD signals centered at the exciton absorption band of 400–450 nm, which precisely mirrored the chiral configuration of the surface ligands. Notably, the chiral CD responses remained invariant upon solution dilution but were substantially attenuated in drop-cast films. Such concentration-independent chiral behaviors exclude the origin of interparticle aggregation-induced chirality. Instead, the chiral optical activity originates from direct electronic coupling between the molecular orbitals of chiral ligands and the excitonic wavefunctions of the perovskite lattice as shown in Figure 3d down panel, which effectively imprints molecular chiral information onto the delocalized electronic states of the semiconductor. This work first demonstrated the realization of exciton-level chiral optical activity via interfacial electronic state coupling in colloidal perovskite nanoplatelets.

Furthermore, the strength of such interfacial chiral transfer is highly tunable by the electron-donating capability of surface capping ligands. Variations in ligand electron-donor ability modulate the orbital overlap degree and electronic coupling efficiency between chiral ligand frontier orbitals and perovskite excitonic states. Ligands with stronger electron-donating characteristics facilitate more intensive interfacial electronic interaction, amplifying the chiral perturbation imposed on the perovskite band-edge states and thereby enhancing the magnitude of CD responses. In contrast, weak electron-donor ligands yield insufficient orbital hybridization, restrict chiral information injection, and ultimately weaken the overall chiral optical activity of perovskite nanoplatelets. Such a ligand-governed coupling mechanism provides a facile and effective strategy to precisely modulate the chiral amplitude of low-dimensional perovskites at the electronic-state level (Dunlap-Shohl et al., 2024).

#### 2.2.2 Intrinsic chiral PNCs constructed via dimensional regulation

Asymmetric structural centers, as the essential source of polarized luminescence in halide perovskite systems, are primarily generated through two distinct material engineering routes: extrinsic ligand-induced surface symmetry breaking and intrinsic *in-situ* crystallographic chirality construction. In the extrinsic modulation mode, chiral surface ligands drive asymmetric bonding and subsurface lattice reconstruction via noncovalent interactions or defect-site coordination, thereby imprinting molecular chirality onto the achiral inorganic framework. Although such post-synthetic surface engineering can effectively activate chiroptical responses, the chiral distortion is mostly surface-confined and highly susceptible to external disturbances, leading to insufficient stability and limited dissymmetry factors. In comparison, intrinsic chiral perovskites constructed via dimensional regulation achieve long-range lattice symmetry breaking during *in-situ* crystallization. Chiral organic spacers participate in lattice assembly as rigid building units rather than simple capping ligands, realizing robust and scalable chiral integration across the entire inorganic framework (Dong et al., 2026; Jana et al., 2020; Son et al., 2023). The resultant structural asymmetry, combined with the strong spin-orbit coupling inherent to lead halide perovskites, induces exciton fine-structure splitting and nonequilibrium spin redistribution, enabling stable, high-purity intrinsic CPL emission.

**FIGURE 4:**
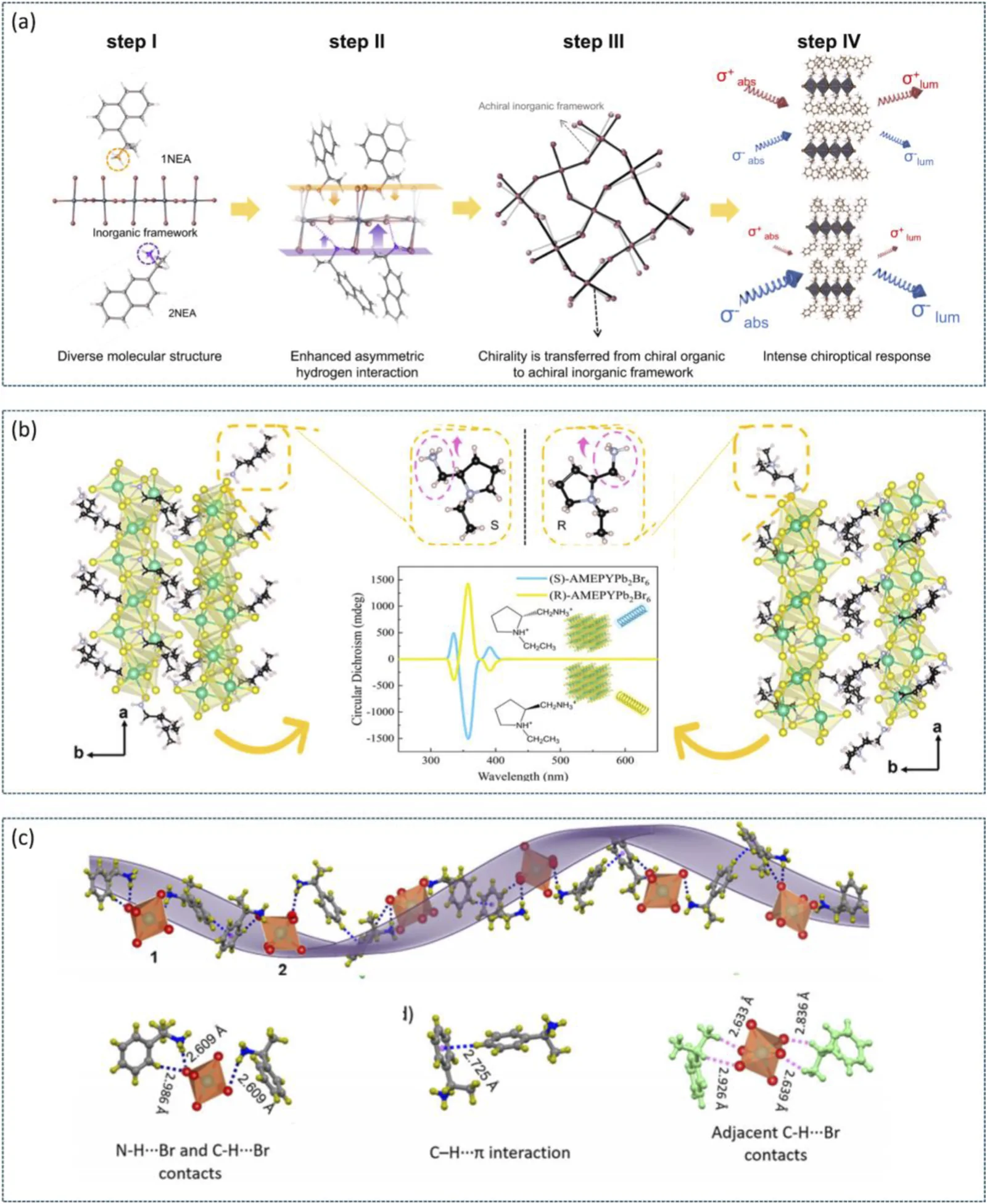
Chiral generation processes and underlying mechanisms in perovskite nanocrystals with varied lattice dimensions. **(a)** Chiral induction mechanism in two-dimensional (2D) perovskite nanocrystals Copyright 2023, Springer Nature (Son et al., 2023); **(b)** Chiral induction mechanisms in one-dimensional (1D) and **(c)** zero-dimensional (0D) perovskite nanocrystals, Copyright 2025, Wiley (Babu et al., 2025; Zheng et al., 2026).

Notably, 2D, 1D, and 0D perovskite crystal structures exhibit distinctly unique chiral induction mechanisms and structural symmetry-breaking behaviors dominated by dimensional confinement and organic spacer–inorganic lattice interactions. In layered 2D perovskites, interlayer chiral aromatic ammonium spacers govern chirality transfer primarily through directional asymmetric hydrogen bonding. Typical chiral NEA^+^ cations establish anisotropic N-H···Br hydrogen-bonding arrays along PbBr_4_ inorganic planes, triggering selective distortion of Pb-Br-Pb bond angles and continuous out-of-plane helical twisting of the layered framework, Figure 4a illustrates the chiral luminescence mechanism of 2D perovskites driven by chiral spacer molecules (Son et al., 2023). The incorporated chiral organic spacers interact with the inorganic lattice via anisotropic noncovalent interactions, triggering subtle lattice distortion and inversion symmetry breaking of the two-dimensional metal-halide framework. This extent lattice helicity completely breaks inversion symmetry and generates prominent Rashba-Dresselhaus spin splitting, providing a reliable spin-asymmetric origin for efficient intrinsic CPL emission.

Differentiated from the planar distortion mode of 2D systems, axially connected 1D perovskite frameworks possess superior structural flexibility for lattice helicalization. The surrounding chiral cations simultaneously integrate directional hydrogen bonding and intermolecular π-π stacking interactions, collectively driving macroscopic helical distortion of face-sharing octahedral inorganic chains Figure 4b, (Zheng et al., 2026). Unlike the distortion-based intrinsic chirality of continuous 2D and 1D perovskite lattices, 0D perovskite chiral clusters rely entirely on organic spacer-guided long-range ordered assembly. Isolated zero-dimensional metal-halide octahedral units exhibit negligible intrinsic lattice distortion, and their macroscopic superhelical architecture is exclusively dictated by bulky chiral organic spacers. During crystallization, anisotropic hydrogen-bonding networks and steric hindrance effects originating from chiral spacers precisely regulate the periodic rotational arrangement of discrete clusters, assembling disordered monomers into long-range ordered superhelical superstructures, Figure 4c (Babu et al., 2025). Such spacer-mediated packing asymmetry breaks the ensemble inversion symmetry, endowing 0D perovskite systems with prominent intrinsic chiroptical activity in the absence of local inorganic lattice distortion. Overall, the dimensionality-dependent topological connectivity of metal-halide frameworks, coupled with the distinct noncovalent modulation modes of chiral organic spacers, dominates the degree of structural symmetry breaking, enabling dimension-tunable intrinsic chiral induction in perovskite nanosystems.

## 3 Realization of polarized emission in aggregated state thin films

The fabrication of high-performance polarized luminescent thin films is a prerequisite for the implementation of polarized perovskite light-emitting diodes (LEDs). The intrinsic TDMs anisotropy and linearly polarized emission of low-dimensional perovskite nanocrystals are defined by their microscopic geometric confinement and lattice distortion. Nevertheless, randomly aggregated nanocrystals typically average out individual dipole anisotropy at the macroscopic scale, resulting in isotropic bulk emission. In this regard, macroscopic ordered assembly of anisotropic perovskite nanostructures (e.g., nanorods, nanowires, nanoplatelets) serves as a pivotal strategy to preserve, amplify, and translate the microscopic polarization characteristics of single nanocrystals into macroscopic film-level polarized luminescence. This section systematically summarizes state-of-the-art macroscopic assembly methodologies for polarized emissive thin films, focusing on their working principles, technical features, applicable material systems, and key figure-of-merit parameters including the DOP.

### 3.1 Macroscopically aligned thin films for linearly polarized emission

#### 3.1.1 Inkjet printing

**FIGURE 5:**
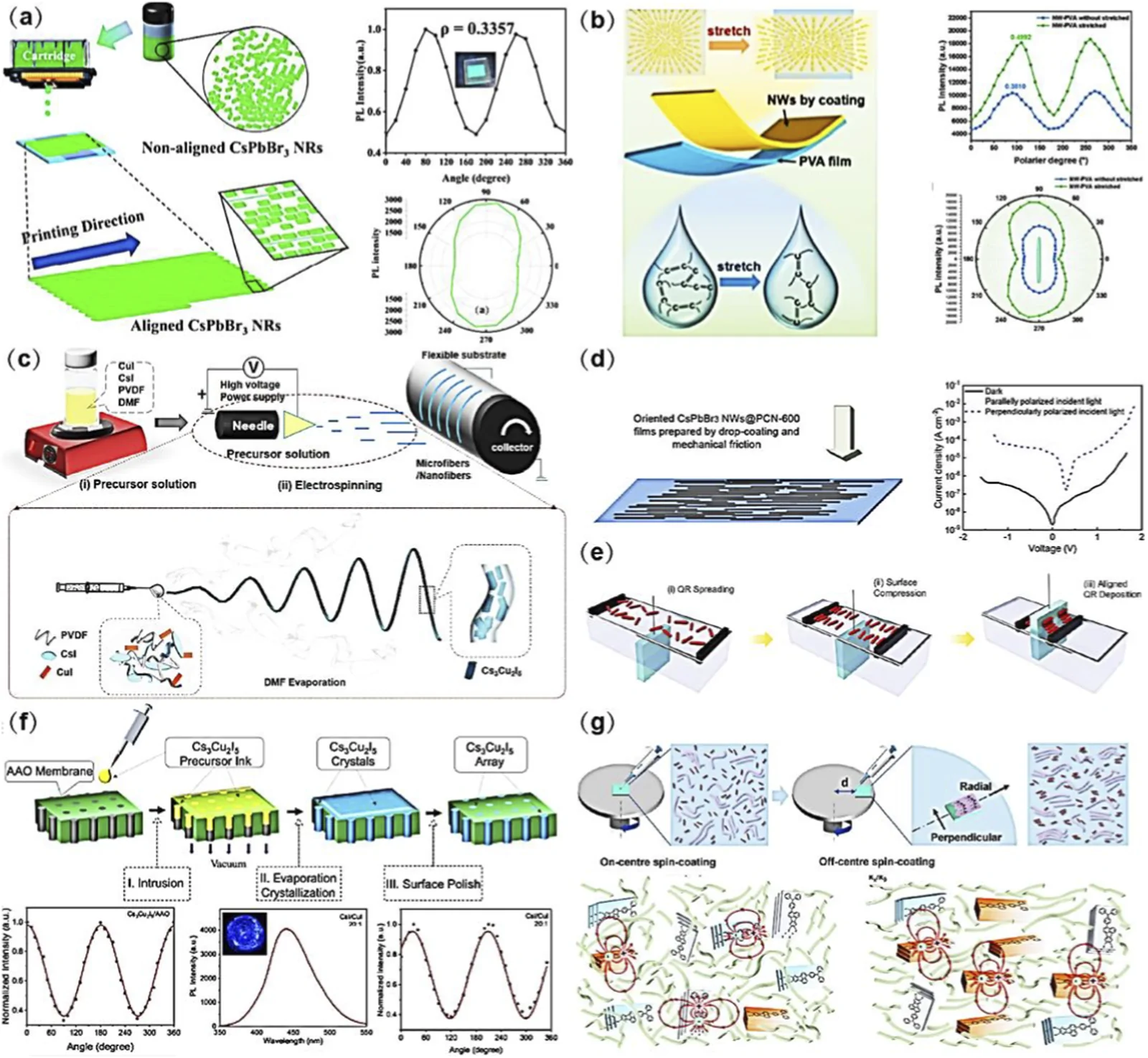
Strategies for fabricating linearly polarized luminescent thin films from linearly emissive perovskite nanocrystals. Fabrication of linearly polarized perovskite nanocrystal films via **(a)** inkjet printing; **(b)** Mechanical stretching; **(c)** Electrospinning, Copyright 2022, American Chemical Society (Jiang et al., 2022); **(d)** Mechanical rubbing, Copyright 2026, Academic Press Inc. (Wang et al., 2026); **(e)** LB technique to prepare oriented nanorod, Copyright 2024, Springer Nature (Ye et al., 2024); **(f)** Template-assisted assembly, Copyright 2024, Optical Society of America (Wang et al., 2024); **(g)** Off-center spin-coating Copyright 2020, Elsevier BV (Wang et al., 2020).

Inkjet printing is a non-contact, digitally programmable deposition technique that enables the scalable fabrication of large-area, uniform polarized luminescent films, exhibiting great potential for integrated multifunctional optoelectronic devices (Gupta et al., 2019). Leveraging fluid shear force and capillary dynamics during ink deposition, this strategy can precisely manipulate the long-range directional alignment of anisotropic nanocrystals, thereby retaining and amplifying their intrinsic TDM anisotropy in solid films. To achieve continuous and large-scale nanocrystal alignment, Zhou et al. developed a continuous-flow contact inkjet printing strategy (Zhou et al., 2019). Different from discrete droplet deposition, the continuous ink stream induced robust hydrodynamic shear and capillary forces at the nozzle–substrate interface, driving quantum rods to spontaneously orient along fluid flow directions and form large-area ordered assemblies. Furthermore, Wang et al. proposed a solute morphology engineering strategy to improve film uniformity and polarization performance (Wang W. et al., 2025). Anisotropic CsPbBr_3_ nanorods with an aspect ratio of ∼2.5 were adopted to replace conventional isotropic nanocubes. The gas–liquid interfacial capillary attraction induced by one-dimensional nanorods effectively suppressed the coffee-ring effect, yielding ultra-flat films with a low root-mean-square roughness of 0.226 nm, Figure 5a. Meanwhile, the hydrodynamic shear force generated by the moving printhead drove unidirectional nanorod alignment along the printing direction. Benefiting from the retained intrinsic TDM anisotropy of oriented nanorods, the printed perovskite LEDs achieved a polarization degree of 0.3357 and a peak luminance of 101 cd m^-2^.

#### 3.1.2 Mechanical stretching

Mechanical stretching is a facile, low-cost, and scalable strategy for constructing macroscopically oriented composite films. By embedding shape-anisotropic nanocrystals within flexible polymer matrices, external tensile strain can induce interfacial shear stress to reorient randomly dispersed nanocrystals along the stretching axis, thereby translating microscopic dipole anisotropy into macroscopic polarized emission (He et al., 2020; Wang J. et al., 2021). Lu et al. integrated *in-situ* perovskite crystallization with controlled mechanical stretching to fabricate highly polarized perovskite–polymer composite films (Lu et al., 2017). CH_3_NH_3_PbBr_3_ precursors were blended with polyvinylidene fluoride (PVDF) in dimethylformamide solution to form uniform precursor films. Controlled triple tensile stretching during film maturation optimized the uniaxial orientation of perovskite grains, achieving a maximum polarization ratio of 0.33 while maintaining a high PLQY of 80%. Zhang et al. further verified the effectiveness of post-synthetic mechanical stretching for polarization enhancement (Zhang et al., 2022). High-quality, phase-pure CsPbBr_3_ nanowires were synthesized via prolonged hot-injection reaction and dispersed into polyvinyl alcohol (PVA) matrices via spin coating. The unstretched composite film exhibited a moderate polarization degree of 0.381, while tensile stretching significantly improved the long-range nanowire alignment density, elevating the macroscopic polarization degree to 0.499, Figure 5b. This improvement confirms that mechanical stretching can effectively suppress random dipole disorder in polymer composites and amplify the intrinsic optical anisotropy of perovskite nanowires.

#### 3.1.3 Electrospinning

Electrospinning is a continuous, high-throughput manufacturing technique capable of constructing one-dimensional fibrous network architectures, which provides natural spatial confinement for the directional assembly of anisotropic nanocrystals (Jiang et al., 2022; Xue et al., 2019). The microscale fiber channels restrict nanocrystal rotation and disordered aggregation, enabling the retention of single-particle TDM anisotropy at the macroscopic network level. Meng et al. realized *in-situ* growth of oriented MAPbBr_3_ nanorods within electrospun PVA nanofibers (Meng et al., 2019). The synergistic quantum and dielectric confinement inside isolated nanofibers endowed the embedded nanorods with prominent optical anisotropy, achieving a single-fiber DOP of 0.53 and a network-film DOP of 0.42. Notably, a positive correlation between nanocrystal aspect ratio and macroscopic polarization performance was clearly established. To address the toxicity and instability issues of lead-based perovskites, Jiang et al. developed one-step electrospun lead-free Cs_3_Cu_2_I_5_/PVDF composite films with blue polarized emission (Jiang et al., 2022), Figure 5c. Precursor concentration modulation enabled precise morphological tuning from microfibers to 35 nm ultrathin nanofibers. The loose spatial confinement of microfibers led to randomly oriented Cs_3_Cu_2_I_5_ domains and limited polarization (DOP = 0.18). In contrast, the stringent nanoscale confinement of ultrathin nanofibers enforced the directional crystallization of high-aspect-ratio (1.52) Cs_3_Cu_2_I_5_ nanorods aligned along the fiber axial direction. The highly oriented nanostructure arrangement maximized dielectric and quantum anisotropy, yielding an improved DOP of 0.4 and a high PLQY of 86.1%.

#### 3.1.4 Mechanical rubbing

Mechanical rubbing is a technique that utilizes directional frictional dragging force to reorganize randomly stacked nanocrystals into unidirectional arrays, which is universally applicable for amplifying film-scale polarized emission. Wei et al. developed highly oriented CsPbBr_3_ nanowire films by deploying a unidirectional brush stroke over drop-cast assemblies (Wei et al., 2020). They evaluated both wet rubbing before complete solvent evaporation and dry rubbing after full desiccation, discovering that the frictionally rearranged nanowire films could be successfully integrated into polarized light-emitting diodes. To further enhance structural ordering and polarization stability, Wang et al. constructed hybrid CsPbBr_3_ nanowire/PCN-600 metal-organic framework (MOF) composite films via a two-step solvothermal method, Figure 5d (Wang et al., 2026). Nanowires were encapsulated within the one-dimensional straight channels of MOF matrices, followed by multi-cycle unidirectional mechanical rubbing. The external frictional force drove the synchronous alignment of MOF channels and embedded perovskite nanowires along the rubbing direction, effectively eliminating isotropic disordered emission. Benefiting from the highly ordered dual nanostructure, the photoluminescence polarization ratio was significantly promoted from an isotropic 0.065 to a highly anisotropic 0.38.

#### 3.1.5 Langmuir-Blodgett technique

Gas-liquid interfaces inherent to the Langmuir-Blodgett (LB) technique offer a thermodynamically ideal platform to drive long-range ordered self-assembly of colloidal nanocrystals. Evaporation-derived capillary forces and interfacial surface tension cooperatively orient anisotropic nanocrystals into densely packed, highly ordered superlattices. This assembly route fundamentally suppresses random particle aggregation that disrupts transition dipole alignment, and thus dramatically amplifies the intrinsic microscopic polarization anisotropy, as illustrated in Figure 5e. Leveraging the unique interfacial regulation capability of LB processing, Ye et al. finely tuned colloidal evaporation kinetics to assemble CsPbI_3_ perovskite nanoplatelets into large-area, well-ordered superlattice films with uniform edge-up stacking orientation (Ye et al., 2024). Benefiting from the preserved and magnified optical anisotropy originating from individual nanoplatelets, the resulting superlattice film delivered an outstanding electroluminescence polarization degree of 0.744.

#### 3.1.6 Template-assisted assembly

Template-confined growth utilizes rigid nanochannel architectures to restrict nanocrystal nucleation and growth, realizing deterministic uniaxial orientation. Wang et al. adopted anodic aluminum oxide (AAO) template nanochannels to confine the growth of lead-free Cs_3_Cu_2_I_5_ nanostructures (Wang et al., 2024), Figure 5f. The rigorous vertical channel confinement produced perfectly aligned nanowire arrays, and the strong dielectric confinement effect between nanowires and template walls retained axial TDM orientation, achieving blue polarized emission with a DOP of 0.50.

Similarly, soft-template-guided directional crystallization was developed to construct oriented MAPbX_3_ triangular nanowire arrays along the [010] crystallographic direction, where template periodicity enabled precise modulation of nanowire spacing and alignment density (Xiong et al., 2024). Beyond conventional templates, boron nitride nanotubes with sub-nanometer confined channels were adopted by Botka et al. to grow ultrahigh-aspect-ratio (>100) perovskite quantum wires with only 1 to 9 unit-cell diameters (Botka et al., 2026). The extreme spatial confinement strictly restricted dipole randomization, locking TDMs orientation along the nanotube axial direction and generating highly line-polarized photoluminescence. Template confinement technique could be combinded with inkjet infiltration, Lin et al. realized vertical ordered assembly of perovskite nanowire arrays (Lin et al., 2020) by combing the above two techniques. Perovskite precursor inks were infiltrated into porous AAO templates under bottom vacuum suction, followed by thermal annealing to induce *in-situ* crystallization of vertically oriented CH_3_NH_3_PbBr_3_ nanowire arrays. The rigid template channels enforced uniaxial nanowire orientation, while the substantial dielectric mismatch between high-permittivity perovskite nanowires and low-dielectric template walls further suppressed out-of-plane electric field components and retained axial TDMs orientation. The synergistic structural and dielectric anisotropy yielded a high linear polarization degree of 0.84.

#### 3.1.7 Off-center spin-coating

By offsetting the substrate from the spin-coater rotation center, the asymmetric centrifugal radial shear force drives fluid-borne anisotropic nanocrystals to orient along radial trajectories, realizing long-range ordered assembly and macroscopic polarization retention (Yuan et al., 2014). Wang et al. optimized the off-center spin-coating process for CsPbBr_3_ nanowire alignment, Figure 5g (Wang et al., 2020). Under optimized conditions (1700 rpm rotation speed, 4 cm eccentric distance, high-boiling toluene solvent), the prolonged fluid shearing window enabled uniform unidirectional nanowire orientation, yielding oriented films with a photoluminescence polarization degree of 0.46.

### 3.2 Chiral assembly and confined growth for chirality-retained circularly polarized emission films

Consistent with the aggregation-state assembly principle for linearly polarized emission, the macroscopic realization of CPL in perovskite films also relies on rationally designed structural assembly and spatial confinement. Microscale perovskite nanocrystals possess intrinsic chiral excitonic properties or chiral-inducible structural features at the single-particle level. However, random aggregation of nanocrystals in conventional film fabrication triggers macroscopic chiral racemization. To address this issue, a series of chiral assembly and template-confined strategies have been developed to lock microscopic chiral configurations during film formation. According to the assembly pathways and chirality-preserving mechanisms, these strategies can be divided into two categories: matrix-mediated chiral assembly and photonic/structural symmetry-breaking confined assembly, as comprehensively discussed below.

#### 3.2.1 Chiral polymer matrix mediated chiroptical amplification

Chiral polymer and supramolecular matrix assembly represents a mainstream solution-processing strategy to preserve microscopic chirality of perovskite nanocrystals during macroscopic film aggregation. Rather than simply introducing exogenous chiral signals, embedding perovskite components within chiral supramolecular and polymeric networks enables efficient interfacial chirality transfer, macroscopic chiral ordering regulation, and chiroptical signal amplification, which fundamentally suppress aggregation-induced macroscopic chiral racemization and realize solid-state CPL retention.

**FIGURE 6:**
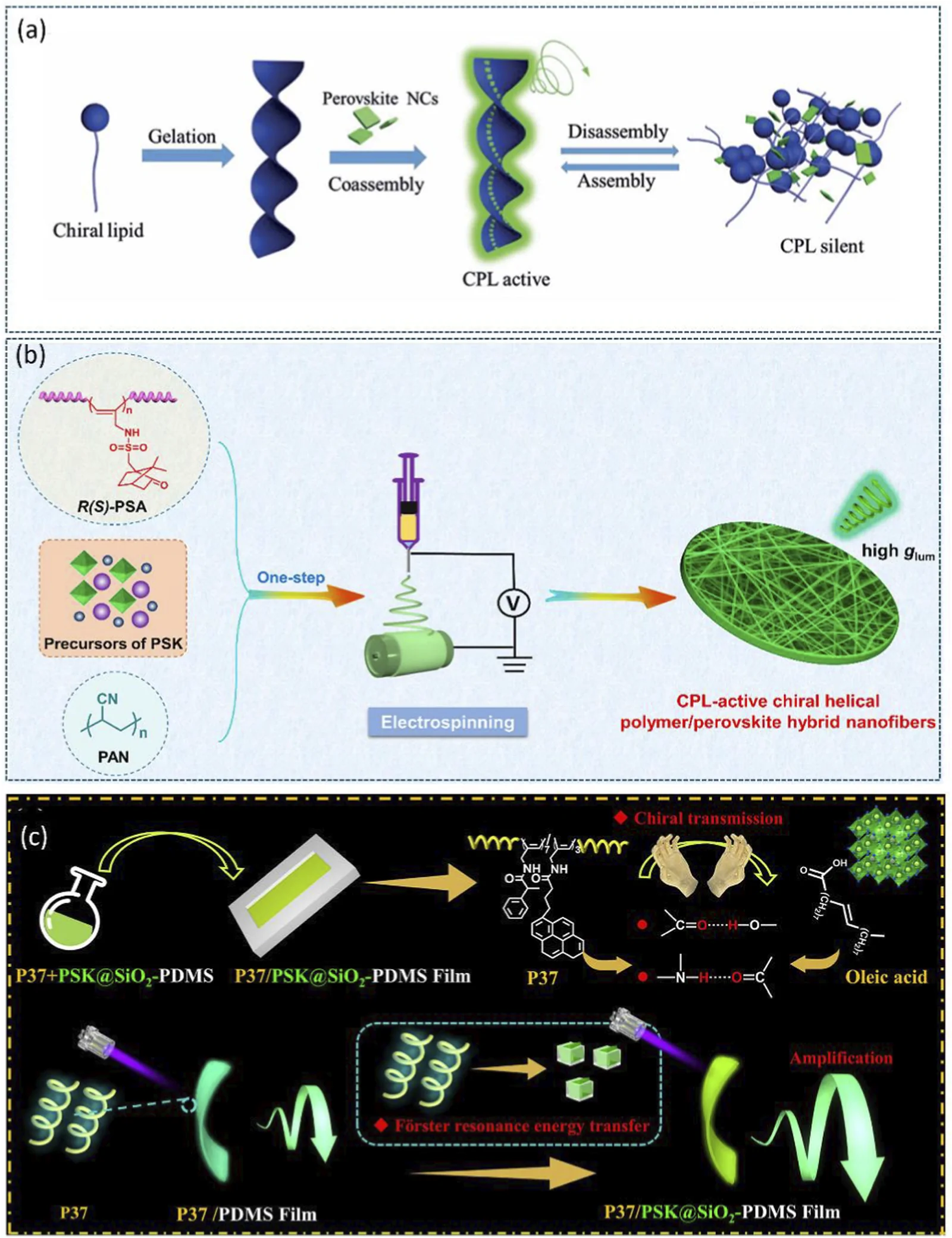
Chiral polymer-assisted polarized luminescence of perovskite nanocrystals in aggregated states. **(a)** Regulation of aggregate rotation orientation via helical molecular networks, Copyright 2018, Wiley (Shi et al., 2018); **(b)** Polarization orientation control through electrospinning strategy, Copyright 2021, American Chemical Society (Zhao et al., 2021); **(c)** Chiral modulation via left-handed polymer blending, Copyright 2025, American Chemical Society (Liu et al., 2025).

Shi et al. utilized a supramolecular co-assembly strategy to blend chiral lipid gelators with CsPbX_3_ nanocrystals in non-polar solvents (Shi et al., 2018). The self-assembled helical organogel network spatially templated and confined perovskite nanocrystals into a persistent chiral packing configuration, successfully prohibiting random chiral disordering and macroscopic racemization during film formation Figure 6a. Such matrix-templated chiral arrangement induced distinct solid-state CPL emission with a *g*_lum_ value of approximately 7.3 × 10^−3^. Similarly, Zhao et al. fabricated hybrid chiral nanofibers by co-electrospinning a mixture of chiral helical polyacetylene and perovskite nanocrystals, Figure 6b (Zhao et al., 2021). The hierarchical fibrous network effectively immobilized perovskite emitters and stabilized chiral microscopic configurations, avoiding racemic chiral rearrangement during solvent evaporation and film curing. This configuration fully exploited the circular-polarization-selective absorption of the chiral polymer matrix to discriminate and retain single-handed circularly polarized emission, achieving intense green and blue CPL emission with absolute *g*_lum_ values of 3.2 × 10^−2^ and 3.0 × 10^−3^, respectively.

In a follow-up study, Liu et al. further developed hydrogen-bond-guided interfacial assembly to construct stretchable multicolor CPL composite films (Liu et al., 2025). Chiral helical polyacetylene (P37) was coupled with partially silica-encapsulated perovskite nanocrystals (PSK@SiO_2_) and embedded within flexible PDMS elastomer matrices, Figure 6c. The exposed oleic acid ligands on PSK@SiO_2_ act as hydrogen-bonding bridging sites, mediating efficient interfacial chirality transfer and chiral fluorescence resonance energy transfer from P37 to perovskite nanocrystals. The robust hydrogen-bonding network stabilizes microscopic chiral configurations and prevents chiral disorder during solidification, realizing intense multicolor CPL emission with a maximum g value of 4.3 × 10^−2^ and a high PLQY of 67%. Notably, reversible CPL intensity modulation can be achieved via strain engineering, with stable performance maintained after repeated stretching-recovery cycles. Moreover, P37 can undergo hydrogen-bond-driven supramolecular helical rearrangement in PDMS, enabling tunable chiral inversion. This work provides a novel design principle for the construction of flexible, stretchable, and multicolor chiral optoelectronic materials.

#### 3.2.2 Chiral liquid crystal and photonic crystal modulation for high-*g*_lum_ CPL in aggregated perovskite thin films

Chiral liquid crystal (LC) and photonic crystal architectures represent advanced photonic modulation strategies that can construct periodic helical microcavities and selective photonic bandgaps. Different from conventional soft supramolecular templating, such photonic microstructure engineering enables resonant amplification of chiroptical asymmetry, breaking the inherent limitation of low *g*_lum_ values in traditional perovskite CPL systems and achieving ultrahigh circular polarization dissymmetry factors close to the theoretical limit. These ordered helical photonic structures eliminate macroscopic optical isotropy via unidirectional spiral arrangement, suppress aggregation-induced chiral racemization, and realize efficient preservation and resonant enhancement of perovskite structural chirality.

**FIGURE 7:**
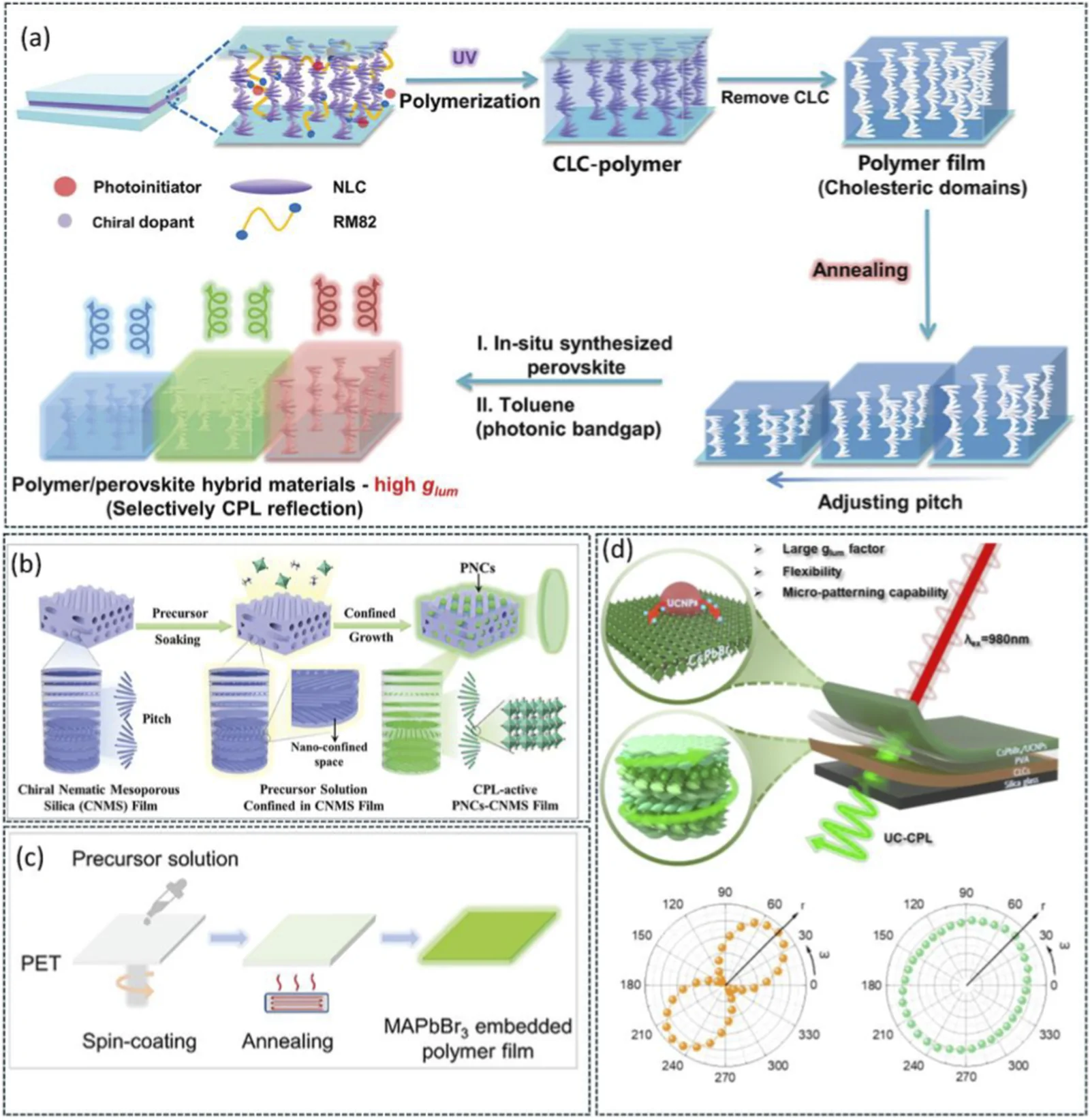
Liquid crystal molecule-mediated chiral regulation of perovskite nanocrystals in aggregated states. **(a)** Amplified chiral emission of perovskite nanocrystal thin films enabled by helical assembly of liquid crystal molecules, Copyright 2024, John Wiley and Sons Inc. (Cao et al., 2024); **(b)** Copolymerization of CLC molecules with perovskite nanocrystals, Copyright 2024, John Wiley and Sons Inc. (Zhang et al., 2024b); **(c)** Fabrication of chiral perovskite quantum dot emissive films via physical stacking of CLC and quantum dot films, Copyright 2024, Wiley (Yu et al., 2024); **(d)** Chiral luminescent thin films based on upconversion perovskite nanocrystals, Copyright 2025, Elsevier (Pu et al., 2025).

Cao et al. fabricated flexible chiral polymer films using cholesteric liquid crystal templates (Cao et al., 2024). Toluene infiltration was adopted to construct well-defined chiral photonic bandgaps, while post thermal annealing precisely regulated the helical pitch to match the emission wavelengths of different perovskite emitters. The resonant spectral matching between photonic bandgap and perovskite emission realized significant CPL amplification, yielding flexible, patternable, and water-stable CPL thin films with an ultrahigh *g*_lum_ value of 0.6, Figure 7a. Zhang et al. designed and synthesized polymerizable CsPbX_3_ (X = Cl, Br, I) perovskite nanomonomers encapsulated with silica shells and functionalized with methacrylate groups, Figure 7b. Via liquid crystal-templated chiral self-assembly followed by *in-situ* photo-crosslinking, luminescent cholesteric liquid crystal elastomer films were fabricated (Zhang et al., 2024b). The as-prepared films exhibit characteristic circularly polarized structural color under ambient light and intense CPL under ultraviolet irradiation, with a maximum *g*_lum_ value of 1.5. Static modulation of CPL intensity and handedness, as well as arbitrary patterning, can be realized by tuning the spectral overlap between the photonic bandgap and photoluminescence peak, alongside the helical chirality of the framework.

For ultrahigh dissymmetry flexible CPL devices, Yu et al. embedded MAPbBr_3_ nanocrystals into polyacrylonitrile films and introduced PEABr passivators to eliminate surface defects, boosting the film PLQY up to 98.3%, Figure 7c (Yu et al., 2024). High-performance flexible CPL composite films were further fabricated by physically stacking the high-brightness perovskite emissive layer with polymerized CLC films, where ITO substrates were employed to optimize liquid crystal molecular alignment. The optimized heterostructure achieved a record-high *g*_lum_ value of 1.81 among state-of-the-art flexible perovskite CPL films. The composite device exhibited outstanding mechanical and environmental robustness, with less than 3% performance attenuation after 1,000 bending cycles at a bending radius of 2 mm, and excellent resistance to water, heat, and UV irradiation, enabling reliable applications in flexible anti-counterfeiting labels. Pu et al. further extended the photonic amplification strategy to upconversion CPL systems (Pu et al., 2025). A one-step *in-situ* synthesis method was developed to prepare composite films integrating CsPbBr_3_ perovskite nanocrystals and NaYF_4_:Yb/Tm upconversion nanoparticles. By stacking the composite emissive film with right-handed cholesteric liquid crystal polymer layers, the matched photonic bandgap at 524 nm enabled selective resonant reflection of upconverted emission. The resulting flexible upconversion CPL device delivered an ultrahigh *g*_lum_ value of 1.80, Figure 7d, which is the highest reported value for perovskite-based upconversion chiroptical systems. The fully solution-processed film retained stable emission intensity after 300 bending cycles, demonstrating superior mechanical flexibility and durability.

#### 3.2.3 Chiral hard-template modulation for high-*g*_lum_ CPL in aggregated perovskite thin films

Hard-template confinement, provide another pivotal route for solid-state chirality retention. Different from soft organic matrix templating, rigid chiral scaffolds and geometry-controlled nanostructure assembly can directly break structural inversion symmetry, lock intrinsic chiral lattice configurations, and fundamentally avoid thermal- or solvent-induced chiral racemization, enabling stable and high-efficiency CPL emission independent of flexible supramolecular networks.

**FIGURE 8:**
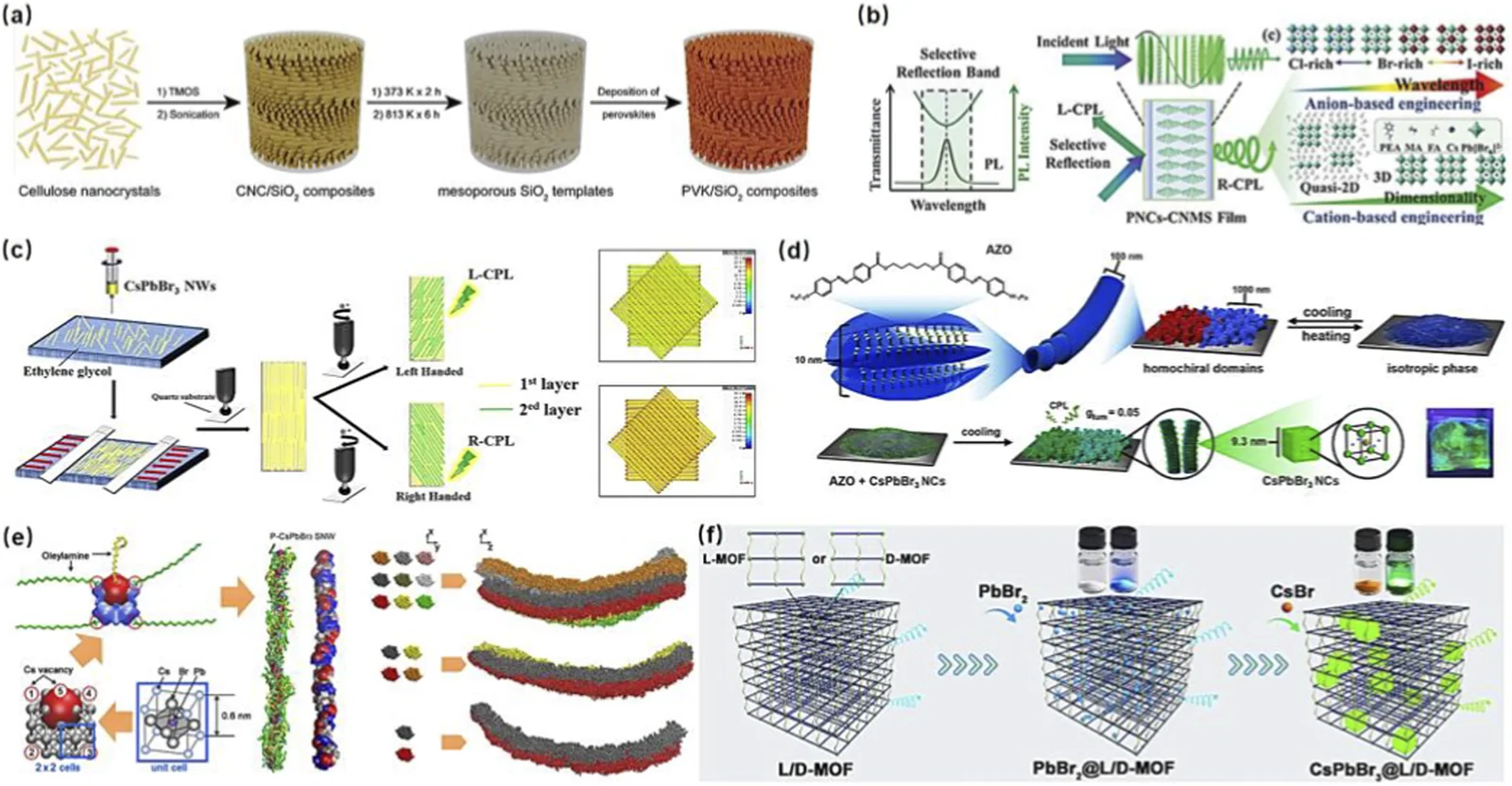
Hard-template strategies for achieving chiral luminescence of perovskite nanocrystals in aggregated states. **(a)** Mesoporous silica template, Copyright 2023, John Wiley and Sons Ltd. (Zhao et al., 2023); **(b)** Modified mesoporous silica template, Copyright 2024, Wiley (Zhang et al., 2024a); **(c)** Self-template assembly of perovskite nanowires, Copyright 2026, Wiley (Peng et al., 2025); **(d)** Anodized aluminum oxide template method, Copyright, 2026 Wiley (Pawlak et al., 2026); **(e)** Intrinsic chirality via helical deformation in all-inorganic perovskite sub-nanowires (<2 nm), Copyright 2025, ACS (Chen G. et al., 2025); **(f)** Metal oxide template strategy, Copyright 2026, Wiley (Ren et al., 2026).

Chiral mesoporous inorganic scaffolds serve as versatile rigid templates for confined chiral crystallization. Zhao et al. first adopted cellulose nanocrystal-derived chiral mesoporous silica (CNMS) as a hard template for perovskite growth (Zhao et al., 2023). CsPbX_3_ perovskites were successfully grown within the chiral mesoporous channels via solution impregnation and *in-situ* thermal annealing, achieving full-color (blue, green, red) CPL emission. Spectral matching between perovskite emission and the intrinsic photonic bandgap of CNMS yielded a prominent *g*_lum_ value of 0.34, Figure 8a. To balance chiral optical performance and luminous efficiency, Zhang et al. optimized the CNMS hard-template system by constructing an inorganic Cs_4_PbBr_6_ passivation layer on *in-situ* grown CsPbBr_3_ nanocrystals Figure 8b (Zhang et al., 2024a). The compact inorganic passivation layer effectively eliminated surface defect states while stabilizing the confined chiral nanostructure. The optimized composite film achieved a steady *g*_lum_ of 0.15 and a greatly improved PLQY of 51.8%. Lu et al. further expanded application potential of the CNMS hard-template strategy (Lu et al., 2024). A series of perovskite systems, including full-spectrum MAPbX_3_, CsPbBr_3_, FAPbBr_3_, and quasi-2D PEA_2_MAPb_2_Br_7_ perovskites, were spatially confined within chiral mesoporous silica nanochannels for regulated crystallization. The rigid chiral confinement effectively suppressed interparticle aggregation and macroscopic racemization, realizing continuous full-color tunable CPL emission with a maximum *g*_lum_ of 0.17. Artificial geometric stacking of anisotropic perovskite nanostructures also enables macroscopic symmetry breaking and strong CPL output. Peng et al. assembled halogen-modulated CsPbX_3_ perovskite nanowires into chiral macroscopic films via precisely designed cross-stacking geometry. By optimizing the stacking architecture to a 4 × 4 layered configuration with a fixed 45° angular offset, macroscopic inversion symmetry was completely broken, and chiral signal cancellation was thoroughly inhibited (Peng et al., 2025). The elaborate nanostructure stacking strategy yielded an ultra-strong CD signal exceeding 3,000 mdeg and stable broadband CPL emission covering 480–800 nm, with a uniform *g*_lum_ of 0.10, exhibiting great potential for multi-level anti-counterfeiting applications, Figure 8c. Pawlak et al. developed a hybrid chiral assembly strategy by doping achiral perovskite nanocrystals into chiral anodized aluminum oxide liquid crystal templates, Figure 8d (Pawlak et al., 2026). The synergistic interplay between nanotube-guided chiral assembly and selective optical filtering contributed to efficient multicolor CPL modulation across red, green, and blue spectral regions, achieving a maximum *g*_lum_ of 0.24. The self-templated approach for perovskite nanowires enables partial Pb-site substitution by target ions to produce nanowires with enhanced circularly polarized luminescence. As shown in Figure 8e, partial surface-site Pb substitution takes place in sub-2 nm sub-nanowires, imparting chirality to the final nanowire products (Chen G. et al., 2025). MOF crystalline scaffolds with intrinsic helical channels also serve as excellent rigid chiral confinement templates. Ren et al. *in-situ* grew CsPbBr_3_ nanocrystals within the continuous chiral helical pores of luminescent L/D-MOFs, constructing CsPbBr_3_@L/D-MOF chiral composite materials, Figure 8f (Ren et al., 2026). The rigid stereoscopic pore structure enabled efficient unidirectional chirality transfer during perovskite crystallization, effectively preventing macroscopic chiral racemization.

In summary, similar to the TDMs orientation assembly mechanism for linear polarized luminescence, all film-scale CPL regulation strategies follow a unified aggregation-state modulation principle: rational chiral templating, spatial confinement, and geometric assembly suppress microscopic chiral disorder and macroscopic racemization, thereby preserving and amplifying intrinsic nanoscale chirality. Soft supramolecular assembly enables facile, flexible, and scalable chiral modulation via interfacial chirality transfer, while photonic crystal resonant amplification and rigid hard-template confinement achieve ultrahigh *g*_lum_ values and robust structural stability via geometric symmetry breaking and photonic resonance. The emerging intrinsic lattice chiral assembly further eliminates the dependence on organic chiral dopants, breaking the performance trade-off between stability, efficiency, and dissymmetry factor. These multi-dimensional assembly strategies effectively bridge microscopic single-nanocrystal chirality and macroscopic film-level chiroptical performance, providing versatile technical support for the development of next-generation high-stability, high-efficiency, and high-dissymmetry chiral perovskite optoelectronic devices.

## 4 Application prospects of PNCs in polarized optoelectronic devices

Previous sections have systematically elaborated on the fundamental mechanisms of polarized emission in perovskite nanosystems, as well as advanced assembly and confinement strategies for retaining microscopic linear/circular polarization and suppressing aggregation-induced chiral racemization in macroscopic thin-film states. Benefiting from their high PLQY, narrow emission linewidth, wide spectral tunability, and full solution processability, assembly-engineered perovskite films enable direct generation of high-purity linearly and circularly polarized light. Different from conventional polarized optical systems that rely on external polarizers and chiral filters to convert isotropic emission-inevitably causing severe photon loss, bulky device configuration, and limited efficiency-perovskite polarized emitters integrate polarization generation and light emission within a single functional layer, offering a compact, high-efficiency, and customizable platform for next-generation polarized optoelectronics. Based on the above structural and chiroptical regulation methodologies, this section systematically summarizes the state-of-the-art applications of perovskite polarized luminescence in display technology, high-security information encryption, polarized photodetection, and spin-optoelectronic devices, and further discusses the technical advantages and practical bottlenecks of such emerging functional systems.

### 4.1 Applications in luminescence devices and advanced display

Three-dimensional display architectures provide highly immersive visual landscapes and represent a major technological frontier for next-generation consumer electronics. Traditional three-dimensional displays typically operate by mechanically filtering unpolarized backlight into orthogonal polarization states, a reductive process that inherently discards at least 50% of the initial photon energy. The capability of tailored perovskite assemblies to directly emit either linearly or circularly polarized light provides an elegant solution for high-efficiency direct polarized illumination. For CPL display applications, Liu et al. constructed a bilayer perovskite-cholesteric liquid crystal composite architecture by integrating MAPbBr_3_ luminescent layers with soft CLC films (Liu et al., 2022).

The synergistic photonic resonance and chiral ordering effect enabled ultrahigh-purity CPL emission with a record-breaking absolute *g*_lum_ factor of 1.9, offering a promising light source for high-brightness glasses-free 3D displays and head-mounted display systems. Beyond photonic template engineering, ligand-mediated morphological assembly further enables the fabrication of integrated chiroptical filter architectures. By regulating solvent evaporation dynamics, enantiomeric R/S-PEABr-capped CsPbBr_3_ nanoplatelets can self-assemble into macroscopically twisted chiral microbelts (Liu H. et al., 2026). The structurally chiral superstructure exhibits broadband CD spanning 200–750 nm, yielding an optimized absorption dissymmetry factor *g*_lum_ of 2.3 × 10^−3^, which is two orders of magnitude higher than that of unassembled nanocrystals. Such self-assembled chiral perovskite microstructures can serve as standalone circular polarization converters, and applicated in future advanced display devices.

**FIGURE 9:**
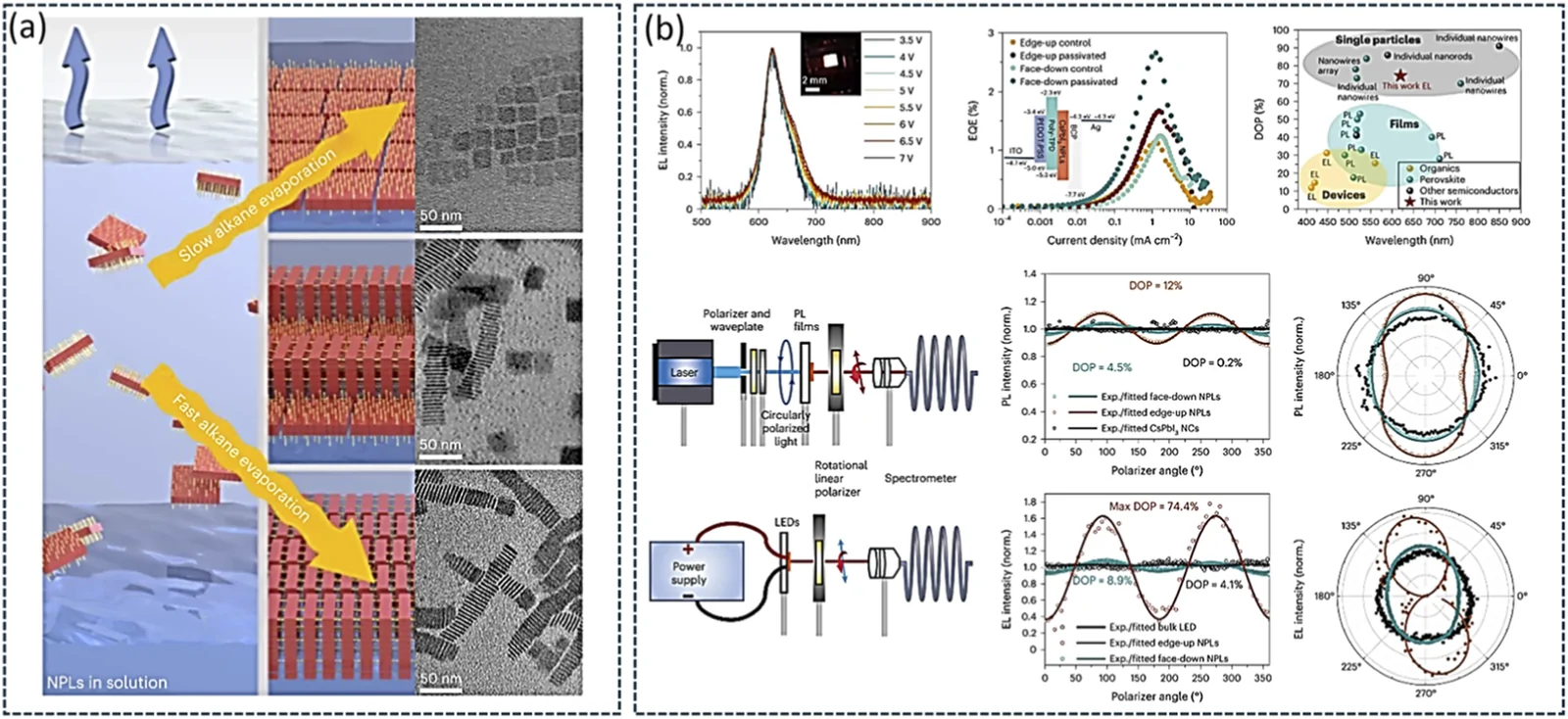
Linearly polarized perovskite nanocrystal light-emitting diodes based on oriented nanoplatelets assembly. **(a)** Modulation of nanocrystal assembly with varied orientation configurations, Copyright; **(b)** Polarized electroluminescence performance of fabricated light-emitting diodes, Copyright 2024, Springer Nature (Ye et al., 2024).

In terms of linear polarization display optimization, orientation-controlled superlattice assembly of two-dimensional perovskite nanoplatelets enables precise modulation of exciton transition dipole orientation. Ye et al. realized face-down and edge-up oriented superlattice films of CsPbI_3_ nanoplatelets via solvent-kinetically controlled self-assembly (Ye et al., 2024). The edge-up configuration induces strong quantum and dielectric confinement, producing exciton fine-structure splitting of 19.77 meV and dominating out-of-plane dipole transition. The corresponding LED device achieves a superior linear polarization degree of 74.4%, Figure 9, representing one of the highest values among external-auxiliary-free polarized light-emitting devices. Meanwhile, the optimized device exhibits ultra-pure red-color coordinates fully compliant with the Rec. 2020 display standard, with external quantum efficiencies (EQE) reaching 2.0% and 2.8% for edge-up and face-down orientations, respectively.

To upgrade commercial LCD backlight systems, shape-anisotropic perovskite nanorods can be macroscopically aligned to construct polarization-enhancing functional films. Song et al. developed a perovskite nanorod alignment enhancement film based on photoalignment technology (Song et al., 2026). The unidirectionally oriented CsPbBr_3_ nanorod arrays emit linearly polarized light with a polarization degree of 0.5. When integrated into LCD backlight modules, the pre-polarized emission matches well with the transmission axis of rear polarizers, increasing photon utilization efficiency from 50% to 75% and boosting overall panel brightness by 25%–30%. Meanwhile, the composite film extends the color gamut to over 95% of the BT.2020 standard, enabling high-color-gamut, thin-form, and low-cost LCD backlight architectures. Composition engineering combined with assembly modulation further improves the stability and polarization performance of perovskite emitters. Jeong et al. synthesized Cs_0.75_FA_0.25_PbI_3_ alloy nanoplatelets with controlled nucleation and growth kinetics (Jeong et al., 2026). The moderate FA^+^ doping induces intermolecular hydrogen-bonding networks, increases surface ligand density, and suppresses non-radiative recombination, elevating the PLQY from 53% to 61% and prolonging the exciton lifetime from 18 ns to 27 ns. The optimized alloy films exhibit excellent phase stability under 40% relative humidity for over 7 days. Moreover, precise aspect-ratio and packing-density regulation of edge-up superlattice assembly enhances the linear polarization degree from 5.1% to 9.4%, demonstrating a feasible strategy for stabilizing and upgrading polarized perovskite optoelectronics.

### 4.2 Applications in the information security and detector fields

**FIGURE 10:**
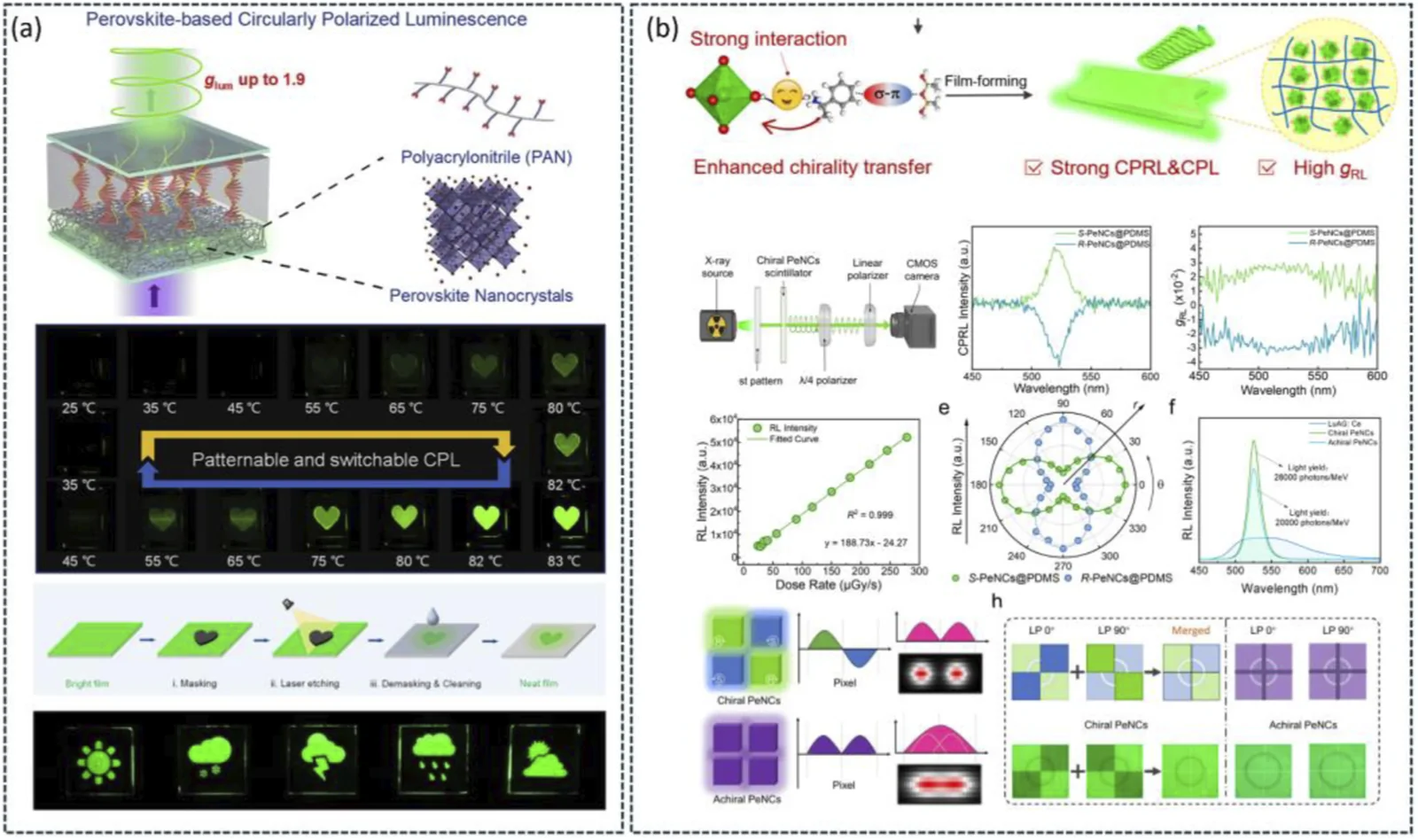
Applications of polarized luminescent perovskite nanocrystals in **(a)** information encryption, Copyright 2025, American Chemical Society (Liu et al., 2022), and **(b)** polarized photodetection, Copyright 2025, American Chemical Society (Wang M. et al., 2025).

Polarized emission and specifically CPL harbor immense application potential within optical communication networks and high-level information security frameworks, a capability rooted in the fact that orthogonal enantiomeric handedness states can serve as a distinct physical medium for high-density information coding. Conventional anticounterfeiting protocols rely on color tunability or variations in luminescence intensity, characteristics that can be readily duplicated or simulated. In sharp contrast, chiroptical anticounterfeiting frameworks rely entirely on the precise polarization state of the emitted photons, which remains completely invisible to the naked eye under ambient conditions and requires specific circularly polarizing analyzers for decoding, thereby offering a significantly elevated tier of cryptographic security. Building upon their previous dual-layer architectural design, Liu et al. fabricated patterned circularly polarized luminescent configurations, utilizing shadow masks to engineer complex luminescent motifs resembling the sun, clouds, and rain that could be selectively revealed or concealed under different circular polarization configurations, Figure 10a (Liu et al., 2022). More importantly, they exploited the intrinsic thermal responsiveness of the host cholesteric liquid crystal matrix, whose helical pitch stretches with increasing temperature to cause a distinct red shift in the corresponding photonic bandgap, to achieve a reversible thermo-responsive cryptographic platform. The active film manifested a high *g*_lum_ asymmetry factor of 1.9 at room temperature, which dropped drastically to 0.1 upon thermal treatment to 83 °C corresponding to the clearing point of the liquid crystal network, and completely recovered its original chiroptical performance upon cooling back to ambient conditions.

To introduce dynamic mechanical encryption, Zhang et al. demonstrated static control over both the emission intensity and circular handedness of CPL by precisely adjusting the blending concentration and helical sense of a chiral dopant identified as R/S 5011 (Zhang et al., 2024b). More importantly, they achieved a reversible mechanically driven chiroptical switch actuated via biaxial tensile elongation. Biaxial stretching compressed the local helical pitch to induce a distinct blue shift in the photonic bandgap, allowing for dynamic control over the spectral overlap between the reflection bandgap and the underlying emission peak to modulate the output CPL intensity, whereas uniaxial stretching caused a total collapse of the helical structure that eliminated the CPL response entirely. Capitalizing on this mechanical behavior, the authors developed a dynamic information encryption and anti-counterfeiting platform where complex geometric patterns or binary codes were embedded directly within the flexible composite films, rendering the encrypted information entirely invisible in the relaxed state and allowing for full cryptographic decryption through a right-handed circular polarizer only when the film was subjected to biaxial tension.

Finally, long-lasting chiroptical states can be engineered without requiring direct ligand-to-crystal chirality transfer. Liu et al. introduced a structural paradigm for generating multicolor circularly polarized long afterglow by co-encapsulating non-chiral CsPbBr_3_ perovskite nanocrystals and blue long-afterglow particles consisting of Sr_2_MgSi_2_O_7_:Eu^2+^, Dy^3+^ within a rigid protective silica matrix via siloxane hydrolysis (Liu Y. et al., 2026). This synthetic route yielded multi-component composite particles where the circular-polarization-selective absorption of an overcoated chiral helical polyacetylene layer successfully generated a blue circular afterglow with a g_lum_ factor of 6.5 × 10^−2^ rooted in the long-afterglow component. Subsequently, this circular phosphoresced light acted as an internal excitation source that transferred the chiroptical information to green, orange, and red-emitting perovskite nanocrystals through highly efficient circularly polarized phosphorescence energy transfer processes including both resonant and radiative energy pathways. This method realized multicolor chiroptical long-afterglow emission based on perovskite composites with *g*_lum_ values reaching the order of 0.01 with a peak value of 2.7 × 10^−2^, enabling the demonstration of multi-dimensional information encryption driven by a combination of time-resolved decay and chiral state coding.

Expanding these chiroptical materials into high-energy radiation sensing, Wang et al. utilized high-brightness chiral CsPbBr_3_ and polydimethylsiloxane composite films as circular polarization scintillators to achieve high-efficiency control over circularly polarized radioluminescence under X-ray excitation Figure 10b (Wang M. et al., 2025). The composite membranes generated intense circularly polarized radioluminescence signals under high-energy radiation with a *g*_lum_ factor of 3.13 × 10^−2^, a value that aligns with the photoluminescence *g*_lum_ factor of 3.13 × 10^−2^. Because circularly polarized emission possesses a distinct propagation angle-dependent asymmetry, arranging adjacent imaging pixels with alternating left-handed and right-handed emitting scintillators provides an effective optical route to isolate and eliminate cross-talk paths through polarization filtering. Experimental evaluations confirmed that these chiral scintillator coatings delivered a spatial resolution of 16.2 lp/mm, heavily outperforming the 8.2 lp/mm baseline delivered by conventional achiral control films to demonstrate extensive utility in high-resolution X-ray imaging platforms. Meng et al. fabricated highly ordered CsPbBr_3_ microwire arrays via template-assisted assembly and further encapsulated the nanostructures with C8TBT organic semiconductors to form robust organic–inorganic heterojunctions (Meng et al., 2026). The one-dimensional geometric anisotropy of microwire arrays enables prominent linear polarization discrimination, delivering a photocurrent dichroic ratio of 1.85. Benefiting from the defect-passivation effect of organic encapsulation, the device achieves an ultrahigh responsivity of 430.6 A W^−1^, exhibiting great potential for high-sensitivity polarized optical signal detection.

### 4.3 Application of chiral PNCs in spintronics

Chiral perovskite assemblies with inherent spin selectivity and circularly polarized electroluminescence capability provide new opportunities for next-generation spin-optoelectronic devices. The chiral-induced spin selectivity effect enables spin-polarized carrier injection and manipulation without external magnetic fields, making perovskite spin-LEDs promising for quantum communication, spin information processing, and chiral quantum photonics. Chiral ligand-modified perovskite nanocrystals enable high-efficiency spin-polarized electroluminescence. He et al. prepared stable chiral CsPbBr_3_ nanocrystals via ultrasound-assisted chiral ligand exchange (He et al., 2025). The fabricated spin-LEDs exhibit prominent electroluminescence dissymmetry factors with g of 2.85 × 10^−2^ (R-enantiomer) and 2.51 × 10^−2^ (S-enantiomer), while achieving high EQEs of 16.8% and 16.0%, respectively. The chiral nanocrystal layer functions simultaneously as the light-emitting active layer and spin-filtering medium, yielding a high internal spin polarization efficiency of 89% and realizing magnetic-field-free spin-polarized light emission. *In-situ* lattice distortion engineering further enhances intrinsic structural chirality and spin selectivity. Yang et al. employed chiral bidentate phosphine oxide ligands to induce permanent helical distortion of surface PbBr_6_ octahedra in MAPbBr_3_ thin films (Yang C.-H. et al., 2024). The locally distorted inorganic lattice endows the entire emission layer with intrinsic chiroptical activity, avoiding severe luminescence degradation caused by full-volume ligand modification. The optimized device achieves an ultrahigh g_lum_ of 0.18 while maintaining an EQE of 3.6%, significantly exceeding the performance of conventional spin-LEDs.

**FIGURE 11:**
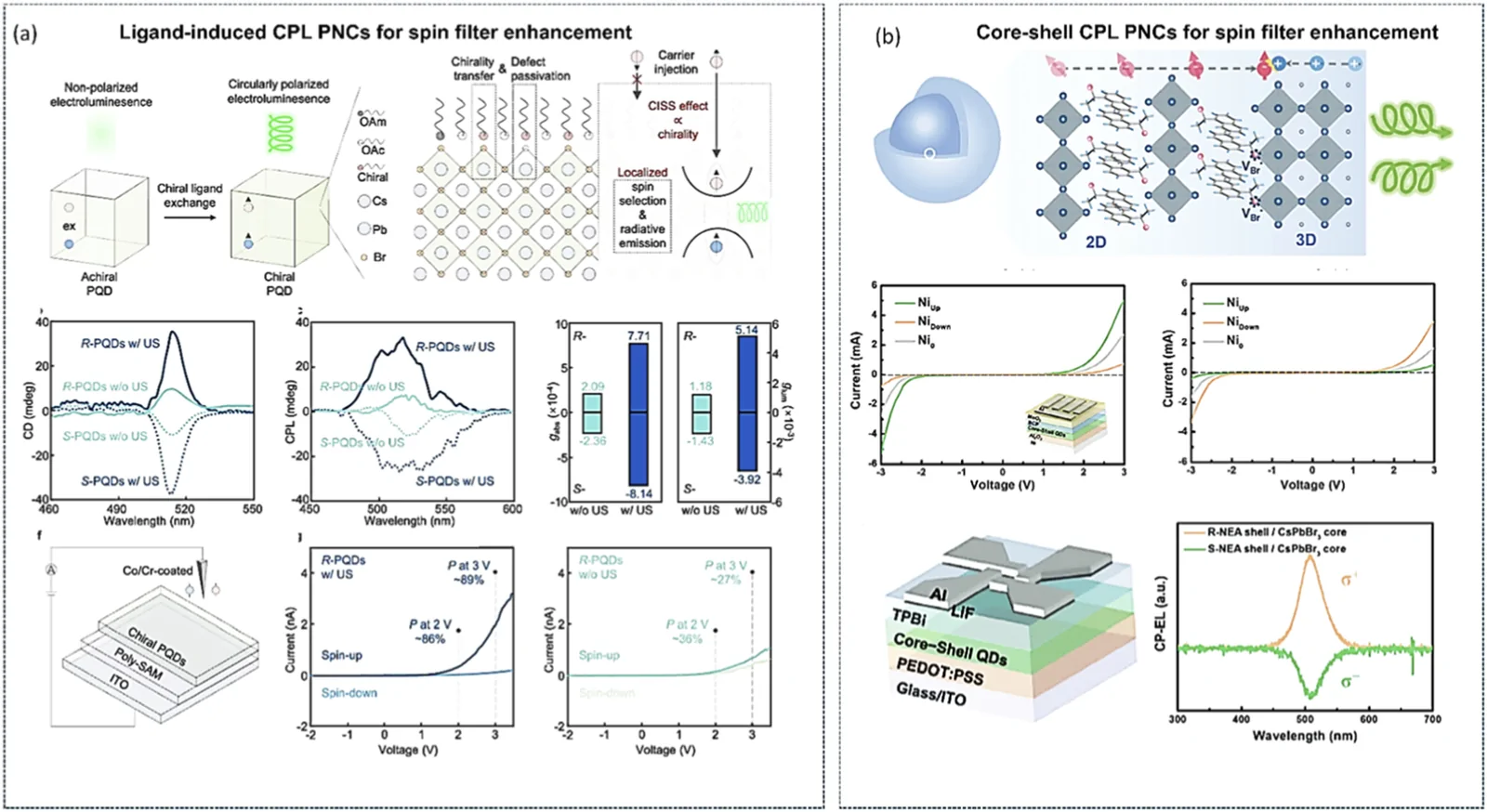
Applications of polarized luminescent perovskite nanocrystals in spintronic devices. **(a)** Circularly polarized perovskite nanocrystals based on surface ligand imprinting, Copyright 2024, Wiley (Yang C.-H. et al., 2024); **(b)** Core-shell structured circularly polarized luminescent perovskite nanocrystals for spin light-emitting devices, Copyright 2024, Wiley (Jang et al., 2024).

Core-shell chiral architecture represents another effective strategy to balance high brightness and spin polarization performance. Jang et al. designed achiral CsPbBr_3_ cores encapsulated by chiral organic perovskite shell layers (Jang et al., 2024). The core-shell structure integrates high radiative recombination efficiency of the achiral core and chiral spin selectivity of the shell, achieving bright green circularly polarized electroluminescence with a polarization degree of 12% (*g*_lum_ ≈ 0.24) and a peak EQE of 5.47% Figure 11b). Moreover, the optimized colloidal ink enables high-resolution inkjet printing of customized spin-polarized luminescent patterns, facilitating scalable fabrication of spin-optoelectronic devices. High-temperature *in-situ* chiral engineering enables simultaneous optimization of quantum efficiency and spin polarization performance. Chen et al. modified CsPbBr_3_ nanocrystals via *in-situ* R/S-bPEABr ligand engineering during crystal growth (Chen D. et al., 2025). The intrinsically chiral perovskite matrix achieves a high PLQY of 89% and a spin polarization efficiency of 88%. The corresponding spin-LEDs deliver a peak luminance of 12,800 cd m^-2^ and an EQE of 15.4%, with a stable circularly polarized electroluminescence factor g of 2.16 × 10^−3^ realizing high-brightness, high-efficiency, and room-temperature-operable spin-polarized light sources.

## 5 Summary and outlook

This review systematically summarizes the latest research progress of perovskite polarized optoelectronic devices, focusing on the core logic of microscopic polarized emission mechanism, chiral regulation strategy, macroscopic ordered assembly, solid-state polarization retention, and multi-functional device application. We first elaborated the fundamental physical origins of polarized luminescence in perovskite systems, distinguishing the anisotropic dipole orientation and geometric confinement-induced linear polarization, as well as the structural symmetry breaking and chirality transfer-enabled circular polarization. The intrinsic mechanisms of linear polarized emission stemming from oriented exciton transition dipoles and low-dimensional structural anisotropy, together with circular polarized luminescence derived from interfacial chiral induction, photonic resonant modulation, and lattice helical distortion, have been comprehensively clarified. On this basis, we further reviewed advanced macroscopic assembly strategies including supramolecular matrix confinement, photonic crystal template symmetry breaking, and ligand-induced chiral self-assembly, which effectively suppress aggregation-induced chiral racemization and dipole disordering, enabling stable retention of high-purity linear and circular polarized emission in solid thin-film states. Benefiting from reliable polarization regulation and solid-state retention capability, perovskite polarized optoelectronic systems exhibit broad application prospects in high-efficiency display backlights, glasses-free 3D displays, high-security information encryption, high-precision polarized photodetection, and magnetic-field-free spin-polarized optoelectronic devices.

Benefiting from superior optoelectronic characteristics including high PLQY, narrow emission linewidth, broad spectral tunability, and excellent solution processability, perovskite-based polarized emitters have overcome the inherent efficiency limitations of conventional polarized optical systems that depend on external polarizers, emerging as a new class of low-cost, high-efficiency, and multifunctional materials for polarized optoelectronic applications. Nevertheless, the industrial translation of perovskite polarized devices remains constrained by several practical bottlenecks, including the unavoidable performance trade-off between polarization purity and luminescence efficiency, unsatisfactory long-term operational stability, immature large-area patterning techniques, and the intrinsic toxicity of lead-based compositions.

In future research, it is essential to further advance the fundamental mechanistic understanding of perovskite polarized luminescence. A systematic and quantitative theoretical framework needs to be established to clarify the coupling between low-dimensional geometric anisotropy, exciton fine-structure splitting and linear polarization purity, as well as the dynamic correlation between chiral structural evolution, interfacial chirality transfer efficiency and circular polarization dissymmetry factors. On this basis, exploring innovative material design routes is critical to address existing performance defects. Specifically, the development of novel low-dimensional lead-free perovskite systems, combined with rational integration with diverse chiral functional components such as chiral molecular complexes and chiral two-dimensional perovskite shell layers, serves as a feasible and effective strategy to comprehensively optimize the comprehensive optoelectronic performance of chiral polarized luminescent materials.

With the continuous innovation of chiral material design principles, gradual improvement of microscopic optoelectronic theoretical systems, and key breakthroughs in large-scale fabrication and encapsulation technologies, the inherent performance trade-offs among polarization degree, dissymmetry factor and luminescence efficiency are expected to be fundamentally resolved. The research paradigm of perovskite polarized luminescence will gradually evolve from empirical performance optimization to precise, mechanism-guided regulation, achieving synergistic improvements in material stability, polarization performance and device practicability. Looking ahead, perovskite polarized optoelectronic technology will occupy a vital position in next-generation high-end displays, quantum optoelectronics, information security and intelligent sensing. It is anticipated to foster disruptive optoelectronic devices and complete industrial ecosystems, while providing solid theoretical and technical support for the upgrading of future information optoelectronic industries.
